# Agronomic performance and remote sensing assessment of organic and mineral fertilization in rice fields

**DOI:** 10.3389/fpls.2023.1230012

**Published:** 2023-10-04

**Authors:** Karen Marti-Jerez, Mar Català-Forner, Núria Tomàs, Gemma Murillo, Carlos Ortiz, María José Sánchez-Torres, Andrea Vitali, Marta S. Lopes

**Affiliations:** ^1^ Sustainable Field Crops, Institute of Agrifood Research and Technology, Amposta, Spain; ^2^ Ministry of Climate Action, Food and Rural Agenda, Lleida, Spain; ^3^ Global Change Unit, Image Processing Laboratory, University of Valencia, Paterna, Valencia, Spain; ^4^ Ente Nazionale Risi, Rice Research Centre, Castello d’Agogna, Italy; ^5^ Sustainable Field Crops, Institute of Agrifood Research and Technology, Lleida, Spain

**Keywords:** rice, nitrogen, nutritional deficiency, remote sensing, Sentinel-2, organic fertilization, precision agriculture

## Abstract

**Introduction:**

Rice heavily relies on nitrogen fertilizers, posing environmental, resource, and geopolitical challenges. This study explores sustainable alternatives like animal manure and remote sensing for resource-efficient rice cultivation. It aims to assess the long-term impact of organic fertilization and remote sensing monitoring on agronomic traits, yield, and nutrition.

**Methods:**

A six-year experiment in rice fields evaluated fertilization strategies, including pig slurry (PS) and chicken manure (CM) with mineral fertilizers (MIN), MIN-only, and zero-fertilization. Traits, yield, spectral responses, and nutrient content were measured. Sentinel-2 remote sensing tracked crop development.

**Results:**

Cost-effective organic fertilizers (PS and CM) caused a 13% and 15% yield reduction but still doubled zero-fertilization yield. PS reduced nitrogen leaching. Heavy metals in rice grains were present at safe amounts. Organic-fertilized crops showed nitrogen deficiency at the late vegetative stages, affecting yield. Sentinel-2 detected nutrient deficiencies through NDVI.

**Discussion:**

Organic fertilizers, especially PS, reduce nitrogen loss, benefiting the environment. However, they come with yield trade-offs and nutrient management challenges that can be managed and balanced with reduced additional mineral applications. Sentinel-2 remote sensing helps manage nutrient deficiencies. In summary, this research favors cost-effective organic fertilizers with improved nutrient management for sustainable rice production.

## 1 Introduction

Rice (*Oryza sativa* L.) is one of the most widely grown crops (Londo et al., 2006) and the staple food for nearly half of the world’s 7.8 billion people (UNSD, 2021). In Europe, rice is mostly cultivated in the Mediterranean countries, with a total harvested area of approximately 608,000 ha, 17% of which is in Spain (FAOSTAT, 2021). The Ebro Delta (Catalonia, NE Spain) is the third largest rice area in Spain with 21.125 ha, representing approximately 19% of the total rice growing area in Spain (MAPA, 2017).

Nitrogen (N) has been a major contributor to crop yield increases since the 1950s, as it is one of the most important limiting nutrients for primary production in many terrestrial ecosystems. Therefore, increased N input often leads to a higher net primary production (Geisseler and Scow, 2014). About 21–25% of the total globally consumed N fertilizer is used in rice crops (Chauhan et al., 2017). Although N application increases rice productivity, poor N use efficiency is a characteristic of irrigated rice systems, mainly due to the rapid loss of applied N via ammonia volatilisation to a greater extent, and also due to surface runoff, leaching, and denitrification (Peng et al., 2006). Furthermore, the long-term application of mineral fertilizers to rice contributes to considerably higher production costs and to environmental pollution by increasing soil acidification, degradation, and compaction of arable soils, thereby restricting future plant growth and yield (Chauhan et al., 2017; Iqbal et al., 2020).

According to the Food and Agriculture Organization (FAO), international fertilizer supplies are likely to remain constrained in the coming years, as stocks are low, and geopolitical tensions have led to additional supply constraints, raising concerns about reduced availability and access to fertilizers (Fixen and Johnston, 2012), which can trigger difficulties in meeting global demands and cause a crisis in food availability (Brunelle et al., 2015). Therefore, further studies are necessary to evaluate sustainable alternatives to mineral fertilizers while improving fertilizer use efficiency through nutritional and agronomic analyses to guide fertilization programs at the field level. These sustainable fertilizer management strategies coupled with improved rice varieties through classic and gene-editing crops (Hu et al., 2022) will ensure improved fertilizer use efficiency and lower environmental impacts in the future. There has been a great interest in organic fertilizers as sustainable nutritional sources to partially replace mineral fertilizers (Singh, 2018) and animal manures have aroused much interest as potential organic fertilizers particularly in regions with intensive livestock farming. In Spain, approximately 40 million tons of pig slurry (PS) are produced annually, as it is the fourth largest pig producer in the world, and the largest in the European Union (approximately 29 million heads per year), contributing approximately 29% of the total production (EUROSTAT, 2023). The use of PS as a nutrient source for rice cultivation could be a form of local supply with high reserves that would allow recycling and reduce management problems associated with increasing local livestock waste. Some studies have reported the potential use of PS as an alternative fertilizer for rice (Pan et al., 2009; Huang et al., 2016; Moreno-García et al., 2017).

Spain also generates large quantities of poultry byproducts as the exploitation of laying chickens reaches approximately 48 million heads per year and is distributed throughout Spanish geography (MAPA, 2022). Several studies have suggested that chicken manure is one of the most efficient manures for rice fertilization (Amanullah et al., 2016; Anisuzzaman et al., 2021).

Organic fertilizers are usually quite low in nutrients, and N release is slow compared to mineral fertilizers (Hirel et al., 2011; Iqbal et al., 2020). Mineralisation and nutrient release rates from organic compounds should be considered for animal manure fertilization because these processes are essential for the availability of nutrients to rice plants (Schmidt and Knoblauch, 2020). Thus, they may not meet the N requirement in a short period for rice growth, especially during the mid to late rice growth period, causing substantial yield losses. It must also be considered that, although it has been extensively reported that the application of livestock manure can have very positive effects on soil fertility (Reimer et al., 2023), its intensive application can pose environmental and human health risks since it has been associated with water pollution, accumulation of potentially toxic elements, pathogen spreading and soil nutrient imbalance (Rathnayake et al., 2023). Exhaustive analysis must be carried out before the manure application and keeping track of the effects of their continuous application on the soil and on the final product that reaches consumer. To achieve a sustainable crop yield without depleting environmental and human impact, it is necessary to fertilise with mineral and manure fertilizers in a coordinated and balanced manner.

The combination of organic and mineral fertilizers could be a better approach to improve and sustain soil fertility and crop production than their application individually, as reported by several studies (Liu et al., 2008; Ding et al., 2018; Iqbal et al., 2020; Anisuzzaman et al., 2021).

The discovery of several cost-effective methods using crop reflectance indexes has greatly facilitated a balanced fertilizer application, while minimizing losses. The Normalised Difference Vegetation Index (NDVI) was developed in the 1970s (Rouse et al., 1973) and evolved into agricultural applications since it can predict plant biomass and nutrient supply (particularly N) by integrating red (RED) and near-infrared (NIR) band information (Pettorelli et al., 2013; Hassan et al., 2019). The Sentinel-2 satellite launched by the European Space Agency in 2016 is a multispectral tool for agricultural practices due to its relatively spatiotemporal high resolution (temporal resolution of five days and spatial resolution of 10m), wide coverage, and availability of 13 spectral bands (ESA, 2023). This tool has also some limitations: i) although the spatial resolution of the Sentinel-2 data constitutes an improvement over other previously available satellites, it is still far from the spatial resolution provided by drone or proximal direct measurements (Mileva et al., 2018; Salgueiro Romero et al., 2020); ii) NDVI saturates when canopies completely cover the soil (particularly under optimal growth conditions) since the red band remains unchanged for dense vegetation conditions, when the leaf area index became high (Pettorelli et al., 2013; San Bautista et al., 2022). In this context, the research presented here explores the use of NDVI to detect deficiencies when values range below saturation observations.

While the general agronomic and physiological effects of organic fertilizers on rice are well understood, variations can occur based on factors such as the type of organic material used, its application rate, soil type, climate, and local management practices. In this context, the research presented herein continues to investigate these complexities to provide more specific recommendations for optimizing the use of organic fertilizers in rice production. Additionally, this study explored the long-term (6 years) impacts on soil composition after continuous applications of the various fertilizer strategies in a rice field. The specific objectives of this study were: (a) Assess the long-term impact of organic fertilization to evaluate local pig slurry and chicken manure as a partial substitute for mineral N fertilizer in rice cultivation; (b) Examine the yield performance and losses to investigate the impact of combined organic fertilization (PS and CM) on rice yield, with specific attention to the observed yield losses as compared to a zero-fertilised strategy; c) Assess safety of organic fertilization by examining the levels of heavy metal contents in rice grains resulting from the different fertilization strategies and ensure they are within safety limits for human consumption; d) Identify nutritional deficiency patterns in rice plants, and assess its effects on yield and yield related traits; e) Evaluate the suitability of NDVI Sentinel-2 data for tracking the nutritional status of rice crops in relation to fertilization strategies, and determine its ability to provide accurate and timely spatial information on crop growth.

## 2 Materials and methods

### 2.1 Experimental fields and climatic characterization

**Figure 1:**
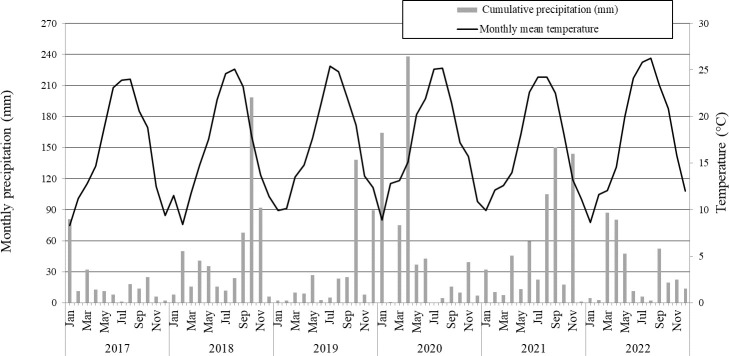
Monthly mean temperature (°C) and precipitation (mm) from 2017 to 2022.

The study was carried out during six growing seasons (2017–2022) in the experimental rice fields of the Ebro Experimental Station at IRTA, located in the municipality of Amposta (40° 41′ 42′′N and 0° 47′ 00′′E) in the Ebro Delta (Southern Catalonia, NE Spain) which is characterised by a Mediterranean climate (**Figure 1**). The mean annual precipitation during the experimental period (2017 to 2022) ranged from 550 mm to 600 mm, mostly distributed during the spring and autumn, showing typical (historical data from 1991 to 2020) precipitation patterns from the region (METEOCAT, 2023). The mean annual temperature between 2017 and 2022 was 18°C with mild winters (mean temperature in January was 9 °C) and hot summers (mean temperature in July was 24°C), as shown in **Figure 1**.

At the beginning of the experiment in 2017, the initial soil texture (USDA, 0–20 cm) was silty clay loam (28.3% clay; 63.2% silt; 8.5% sand), the pH (extract 1:2.5 H_2_O) was 7.94, the electrical conductivity (extract 1:5 H_2_O) was 1.07 dS m^-1^, the organic matter content (Walkley-Black method) was 3.5%, the cation exchange capacity was 12.2 cmol_c_ kg^-1^, the total nitrogen (Kjeldahl method) was 2.0 g kg^−1^, the Olsen phosphorous was 21.4 mg kg^−1^ soil, and the Potassium (ammonium acetate extract) was 154.14 mg kg^−1^ soil.

### 2.2 Experimental design and agronomic management

**Figure 2:**
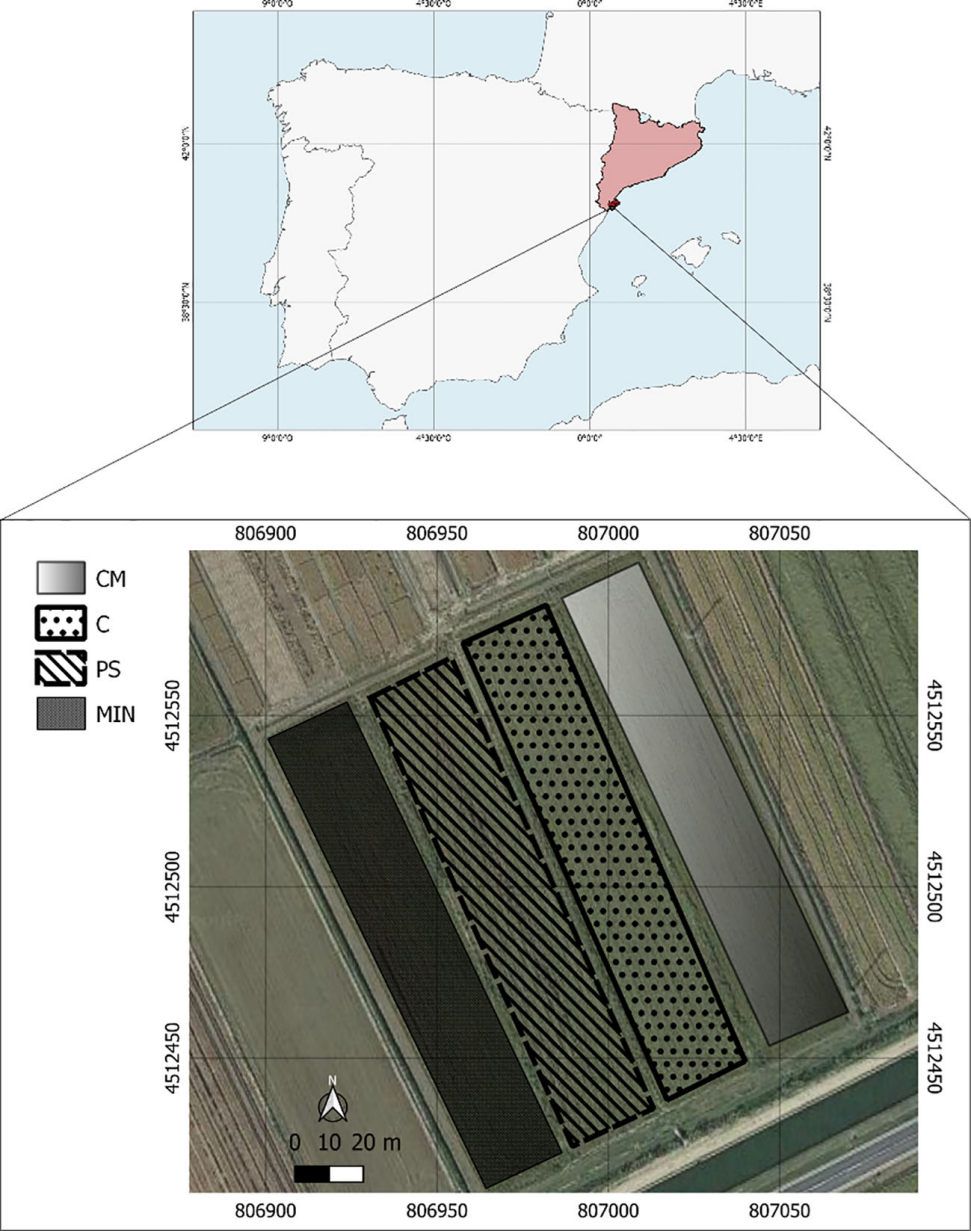
Location of the experimental site and allocation of the four N fertilization strategies (MIN, PS, CM and C) of the trial accomplished from 2017 to 2022. MIN, mineral, PS, pig slurry; CM, chicken manure; C, control.

The JSendra round grain Japonica-type variety was selected as it is widely cultivated in this production area, characterized by high yield and good climate adaptation. It was sown at a seeding rate of 200 kg ha^-1^ between mid-April and mid-May, depending on the annual soil humidity. The experimental design followed a fully randomised block design with four plots treated with four different N fertilization management strategies, which were subdivided into four sub-plots: (1) mineral fertilization (MIN), (2) organic fertilization with pig slurry (PS), (3) organic fertilization with chicken manure (CM), and (4) control without N fertilization (C) (**Figure 2**). Each plot area was 4,270 m^2^ approximately and was divided into four subplots of 1,067 m^2^ approximately.

For all treatments, dry seeding and delayed flooding at the tillering stage (3-4 leaf stage) after the first topdressing application were performed. The field was continuously flooded to a depth of 5–10 cm throughout the growing season.

**Figure 3:**
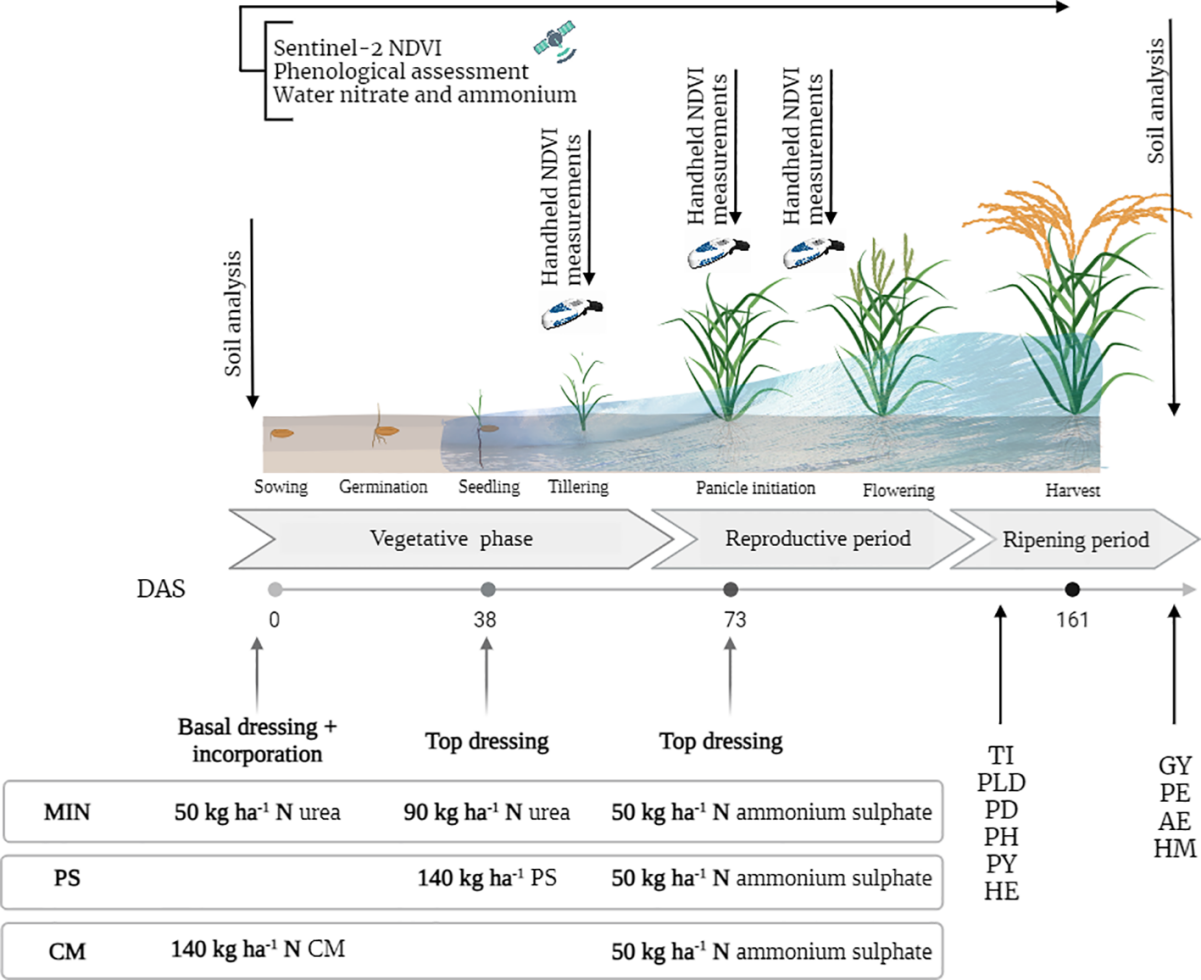
Amount (kg N ha^-1^) and timing of the target N rates applied in the different treatments and assessment schedule according to rice phenological stage and approximate days after seeding (DAS). MIN, mineral fertilizer; PS, pig slurry; CM, chicken manure; DAS, days after sowing; TI, tillering capacity; PLD, plant density; PD, panicle density; PH, plant height; PY, Pyriculariosis; HE, Helminthosporiosis; GY, Grain yield; PE, panicle efficiency; AE, agronomic efficiency; HM, heavy metal concentration in the grain.

The treatment strategies are shown in **Figure 3**. A total of 190 kg N ha^-1^ was applied to all treatments. In the MIN treatment plots, the total N rate was split into three applications: 50 kg N ha^-1^ at pre-sowing, 90 kg ha^-1^ at the tillering stage (3-4 leaves stage), and 50 kg ha^-1^ at panicle differentiation. Urea was used for the first two applications, whereas ammonium sulphate was used for the last application. Pig slurry was applied at the rice-tillering stage immediately before flooding, whereas chicken manure was applied before seeding and incorporated into the soil with tillage at a rate of 140 kg N ha^-1^. Slurry and manure were sampled a few days before application to the farm stock, and using the values derived from the analysis, the amount to be applied was calculated to reach 140 kg N ha^-1^ in all organic fertilization treatments (see **Table 1** for total amounts applied per year). Samples of manures were taken on the same day of application to validate the N applied rates. In PS and CM, organic fertilization was implemented with mineral fertilization at panicle initiation at 50 kg N ha^-1^. To avoid P and K deficiencies in the MIN and C plots, basal K and P fertilizers were applied (50 kg ha^-1^ P_2_O_5_ in calcium superphosphate and 30 kg ha^-1^ K_2_O in potassium sulphate) on the same day as the basal N fertilizer and then incorporated using a rotovator.

**Table 1:** Nutrient amount of pig slurry and chicken manure applied from 2017 to 2022.

Fertilization	Year	Total N applied (kg ha^-1^)	Ammonium NH_4_^+^ – N (kg ha^-1^)	N organic (kg ha^-1^)	P_2_O_5_ total (kg ha^-1^)	K_2_O total (kg ha^-1^)
CM	2017	178.59	31.19	147.40	153.68	163.55
	2018	155.09	105.06	50.03	324.23	181.45
	2019	153.60	99.98	53.61	172.55	104.68
	2020	156.68	101.07	55.62	159.27	98.78
	2021	160.76	115.18	45.57	136.53	121.74
	2022	140.40	50.31	90.09	138.54	119.95
*PS	2017	192.75	126.28	66.47	119.83	113.31
	2018	178.54	142.83	35.71	74.41	111.38
	2019	191.66	92.25	99.41	252.95	110.34
	2020	97.26	59.13	38.20	63.51	74.06
	2021	133.26	96.48	36.78	84.39	87.91
	2022	135.61	89.29	46.32	76.28	118.74

*PS, Pig slurry; CM, Chicken manure.

After harvesting, as is typical in this area, winter flooding with straw addition through puddling was applied until the beginning of January. A cultivator was used for seedbed preparation in March, after which the fertilizers were applied. Seeding was performed under dry soil conditions with a combined seeder and power harrow for fertilizer incorporation.

### 2.3 Crop yield and agronomic traits

Plant and panicle density were determined by scoring six times in squares of 0.25 m^2^ per sub-plot. Height was determined by measuring the height of the plant at the same location on the same day. Two main fungal diseases (*Pyricularia grisea* and *Helminthosporium oryzae*) were assessed using the IRRI Standard Evaluation System to ensure that crops were not severely affected (IRRI, 2002).

Grain weight was determined by harvesting the sub-plots separately using a Mitsubishi trial harvester once full maturation was reached. Once harvested, the levels of heavy metals in the grain were determined for 2021 to evaluate the long-term effects of the different fertilizer strategies on metal accumulation in the final crop product.

Nitrogen Agronomic efficiency (AE) was calculated as described by Cassman et al. (1998).


(2) $$AE\;\left(kg\;grain\;kg^{-1}\;N\;applied\right)=\frac{Y_{NRich}-Y_{N0}}{N_{fert}\;}$$

Y_NRich_ is the grain yield in N fertilised plots, Y_N0_ is the grain yield in no-fertilised plots and N_fert_ is the quantity of N fertilizer applied.

AE_NT_ was calculated by considering the total nitrogen applied. AE_NH4_ was calculated considering only the inorganic nitrogen applied.

In 2021, the grain samples were sent to an external laboratory (Eurofins Agroambiental, S.A., Lleida) to determine the concentrations of the principal heavy metals in the rice grain. The principal heavy metals analysed in the rice grain were the following: arsenic (As), cadmium (Cd), chromium (Cr), nickel (Ni), mercury (Hg), lead (Pb), copper (Cu) and, zinc (Zn). The methodology used is based on the UNE EN 16943 and consists of an acid digestion in a microwave oven and subsequent reading in spectrophotometer (ICP-OES ICAP 7400, Duo Thermo Fisher Scientific Inc., Carlsbad CA, USA), with a high-performance solid-state Chip CID86 detector.

### 2.4 Soil, water, and manure analysis

Soil samples were obtained before sowing and fertilization application, and before the first top-dressing application. By using an auger, 10 soil subsamples were randomly taken from each of the four plots of each treatment with a maximum depth of 20cm. These subsamples were homogenized, kept closed in resistant plastic bags and stored in the refrigerator (approximately 4°C) till the analyses. One kilogram of each plot was sent to an external laboratory (Eurofins Agroambiental, S.A., Lleida) to determine soil moisture (gravimetry 105°C), soil organic carbon (Walkley and Black, 1934), nitrate and ammonium, total nitrogen (Kjeldahl, 1883), total and available phosphorus (Olsen, 1954) and potassium (ammonium acetate extract, spectrometry ICP-OES) concentrations. This procedure was performed for each year of study.

The nutrient composition (ammonium, total N, P_2_O_5_ and K_2_O) of pig slurry and chicken manure was also determined by an external laboratory (Eurofins Agroambiental, S.A., Lleida) each year before their application to calibrate the machinery to apply the target N rates. Owing to the difficulty of the application system, the actual amount applied fluctuated slightly over the years (**Table 1**). Exceptionally, in 2020 the PS application resulted in a lower total N rate compared to the other years due to a considerable difference of the N concentration results between the previous PS analysis and the application day PS analysis. Probably, the pig slurry was not properly homogenized when taking the previous samples, since in the slurry tank there is sedimentation and stratification of nutrients that, in case of not being adequately homogenized, the results of the analysis can be altered and unreal, causing errors in the subsequent application rates.

Nitrate and ammonia contents in the water of the arable land (30 cm depth) and phreatic water (60 cm depth) were assessed using a Nitrachek 404 Meter and MQuant Ammonium Test Supelco. One sample was taken per sub-plot at both depths on different dates. Sampling began at flooding time when water appeared at the studied depths. Samples were taken at approximately 2, 5, 12, 30, 60 and 90 days after flooding each year to obtain the nitrogen leaching profile for each strategy throughout the entire growth cycle since flooding fields. The sampling frequency was higher in the period close to flooding since it is when higher leaching rates and differences between treatments are observed due to the proximity to the fertilizer application (top-dressing 1).

### 2.5 Handheld and satellite sensors

The NDVI was measured at ground level using a GreenSeeker™ (505 hand-held optical sensor, N-Tech Industries, Ukiah, CA, USA). This optical sensor emits brief bursts of red and near infrared (NIR) light, measures the amount of each type reflected back from the plant and displays instantly the measured value in terms of an NDVI reading. The NDVI was calculated using the following equation (Rouse et al., 1973).


(1) $$NDVI=\frac{NIR-RED}{NIR+RED}$$

Two NDVI measurements were taken with the handheld sensor from each sub-plot at approximately 50 to 60 cm above the canopy surface (with a 25 cm major axis oval field of view). Measurements were taken at three main phenological stages of interest for crop nitrogen status assessment: tillering stage, after applying the first top-dressing and representative moment of vegetative development; panicle initiation stage, time of second top-dressing and determining moment for panicle formation and yield; 15 days after panicle initiation, key moment for panicle development and yield (Tu et al., 2019). The NDVI measurements taken at ground level with the handheld sensor were used to validate the satellite-derived NDVI measurements taken with Sentinel-2 through a correlation analysis.

Sentinel-2 level L1 satellite multispectral images were downloaded from SciHub Copernicus for all the years. An atmospheric correction algorithm (LaSRC, Land Surface Reflectance Code) was applied (Vermote et al., 2016). A cloud mask was applied to the reflectivity data images, and the mean and standard deviation of the surface reflectivity of all pixels within each plot were calculated for all Sentinel-2 spectral bands. Subsequently, the satellite derived NDVI was calculated following Equation (1).

The images were processed using the open-source software QGIS ver. 3.28.2 (QGIS Development Team, 2022). NDVI data from Sentinel-2 multispectral images was extracted. Measurements were taken at the same moments as with the handheld sensor at three main phenological stages of interest for crop nitrogen status assessment. These measurements were used to validate the satellite-level data through correlation analysis with the ground-level data. NDVI measurements were also taken throughout crop development all years of study to assess crop spectral response to N fertilization strategy. Only optimal satellite images (without cloud or related problems) were taken. The borders of all the plots were discarded, taking only central values of each one. Non-representative areas of the plots that were affected by other uncontrollable factors not related to N fertilization strategies were also discarded (unusual severe disease affectation and presence of clouds in the plot area). A total of 19 to 22 pixels were obtained from each plot (except for MIN 2021 where only 11 pixels were obtained due to presence of clouds). The rate of senescence (RS) for the plants in each treatment was calculated from the slope of the linear regression equation for the decline in satellite derived NDVI against thermal time (°C) (Pinto et al., 2016).

### 2.6 Statistical analysis

Statistical analyses were performed using JMP 16 (SAS Institute Inc., 2020–2021). Analyses of variance were conducted to determine the effects of treatments, years, and their interactions (considering both factors separately) on yield and yield components, agronomic efficiency, NDVI values, long-term progression of nitrate, ammonium, available phosphorus, and potassium concentrations in soil using the general linear model procedure (GLM). Analyses of variance were conducted to determine the effects of treatments per year (one-way ANOVA) on yield and yield components, agronomic efficiency, grain heavy metal concentrations, soil properties, NDVI measurements, and water nitrate and ammonium concentrations using the general linear model procedure. Multiple comparison mean analysis between treatments was performed using Tukey’s test (HSD) at *p* = 0.05. Moreover, two-way (year and fertilizer strategy) ANOVA analysis was performed by including data from all years. Linear relationships between yield and yield components, satellite derived NDVI, RS, days to maturity and yield were evaluated using the Pearson correlation coefficient (r) (p<0.05). Linear regression analysis was performed to assess the relationship between the ground-based and satellite-based NDVI. For this analysis, only valid Sentinel-2 data were taken, excluding non-valid images with the presence of clouds and that did not coincide in date with the measurements at the ground level. The coefficient of determination (R^2^) was considered to judge the strength of relationship.

## 3 Results

### 3.1 Long -term (6 years) effects of two organic fertilization strategies on rice yield components, agronomic traits, leaf diseases and grain heavy metal accumulation

Over a six-year period, using combined fertilization methods led to a decrease in crop yield by 13% for pig slurry and 15% for chicken manure when compared to using mineral fertilizers alone (see **Table 2**). As anticipated, all fertilization approaches improved crop yield compared to crops that did not receive nitrogen fertilizers (MIN: 134%, PS: 103%, CM: 100%).

**Table 2:** Average (6 year) yield components, agronomic traits and disease scoring of the different fertilization strategies.

Year	fertilization	Yield (Mg ha^-1^)	Plant density (plant m^-2^)	Panicle density (panicle m^-2^)	Tillering capacity (tiller plant^-1^)	Panicle efficiency (yield/panicle)	Plant height (cm)	Pyriculariosis IRRI scale (0-9)	Helminthosporiosis IRRI scale (0-9)
Average	MIN	7.36 ± 0.24 A	179 ± 19.4 A	264 ± 8.0 A	2.2 A	28.8 A	72.9 ± 1.1 A	3.7 A	2.5 C
	PS	6.40 ± 0.22 B	161 ± 17.3 AB	219 ± 6.3 B	2.0 AB	29.9 A	69.8 ± 1.1 B	3.1 AB	2.7 BC
	CM	6.29 ± 0.26 B	178 ± 21.2 A	208 ± 5.4 B	1.9 AB	30.5 A	68.3 ± 1.3 B	3.2 AB	3.2 AB
	C	3.15 ± 0.21 C	157 ± 18.5 B	160 ± 7.1 C	1.5 B	20.1 B	62.6 ± 1.1 C	2.8 B	3.4 A
Pearson correlation (r) Yield			-0.04	0.62***	0.16	0.75***	0.46***	0.02	-0.06
Sources		p(F)							
fertilization		0.000	0.001	0.000	0.000	0.000	0.000	0.007	0.000
Year		0.000	0.000	0.005	0.000	0.000	0.000	0.000	0.000
fertilization × Year		0.100	0.055	0.000	0.000	0.089	0.000	0.000	0.000

MIN, mineral fertilizer; PS, pig slurry; CM, chicken manure; C, control. Measurements are shown as mean ± Standard Error (SE). Results of two-way (top of table, year and fertilization) and one-way (fertilization) ANOVA analysis. Different uppercase letters indicate significant differences at p<0.05 between treatments (two-way ANOVA), as determined using Tukey’s test (HSD). ns, no significance, *p<0.05, **p<0.01, *** p<0.001 according to two-way (year and fertilization strategy) or one-way (fertilization) ANOVA test. Linear relationships between the studied variables and yield were evaluated by the Pearson correlation coefficient (r) (p<0.05). Two-way (year and fertilizer strategy) ANOVA p-value results are shown below the table, including data from all years.

During the early stages of the crop cycle, plant density was greater in the MIN and CM treatments compared to the non-fertilised treatment (an increase of 14% and 13%, respectively). However, the PS treatment showed a plant density similar to that of the non-fertilised treatment (refer to **Table 2**). The higher panicle density achieved with MIN was directly linked to higher yield (correlation coefficient, r=0.62, *p<0.0001*; see **Table 2**). Additionally, MIN yields were positively correlated with plant height, which was 5%, 7%, and 16% higher than PS, CM, and the control (C), respectively. The two organic fertilization strategies showed similar results in terms of yield and yield components.

In terms of fungal diseases, MIN led to a 32% increase in the incidence of *Pyricularia oryzae* infection compared to the control. Intermediate infections were recorded in plots with organic fertilization. Conversely, *Bipolaris oryzae* attacked crops in the CM and control treatments more than those in the MIN and PS treatments.

The differences in yield, plant density, and panicle efficiency between fertilization methods remained consistent across the years, as indicated by the lack of significant interactions between fertilization and year (**Supplementary Table 1**). For most other agronomic parameters, significant interactions were observed between fertilization and year (**Table 2**). In most cases, these interactions indicated that the distinctions among fertilization methods were not significant in all years. Notably, differences among organic and mineral fertilization methods were not significant in 2017, 2018, and 2020, with only the control (C) treatment showing the lowest scores (refer to **Supplementary Table 1**).

When relying solely on mineral fertilization, the crop achieved the highest agronomic efficiency concerning total nitrogen (AE_NT_). In contrast, the utilization of pig slurry and chicken manure led to a notable reduction in AE_NT_, specifically by 27% and 31%, respectively, in comparison to the mineral fertilization approach (MIN) (see **Table 3**). It’s worth noting that the agronomic efficiency linked to ammonium (AE_NH4_) showed no significant variation among the different fertilization methods (refer to **Table 3**).

**Table 3:** Fertilization strategies and year effects using one-way (fertilization strategy) and two-way (fertilization strategy and year effects) ANOVA analysis (Average).

	Fertilization	Average	2017	2018	2019	2020	2021	2022
		***	*	*	ns	*	***	ns
AE_NT_ (kg grain kg^-1^ N applied)	MIN	22.2 A	23.2 a	23.3 a	17.2 a	24.5 a	24.1 a	20.9 a
	PS	16.3 B	13.9 b	15.6 b	12.7 a	22.4 a	14.4 b	18.7 a
	CM	15.3 B	15.2 b	21.0 a	9.5 a	13.8 b	12.5 b	19.5 a
		***	***	*	ns	*	***	**
AE_NH4_ (kg grain kg^-1^ NH_4_^+^ – Napplied)	MIN	22.2 A	23.2 b	23.3 ab	17.2 a	24.5 ab	24.1 a	20.9 b
	PS	23.8 A	19.2 b	18.5 b	21.5 a	30.2 a	18.0 b	35.5 a
	CM	24.1 A	42.9 a	27.8 a	12.9 a	18.9 b	15.9 b	25.9 b

Means separation tests per year and across years (Average) are shown. M, mineral fertilizer; PS, pig slurry; CM, chicken manure; C, control. Measurements are shown as means. Different lowercase letters indicate significant differences at p<0.05 between treatments (one-way ANOVA), as determined using Tukey’s test (HSD). Different uppercase letters indicate significant differences at p<0.05 between treatments (two-way ANOVA), as determined using Tukey’s test (HSD). ns, no significance, *p<0.05, **p<0.01, ***p<0.001 according to two-way (year and Fertilization strategy) or one-way (Fertilization) ANOVA test.

After six years of fertilization, the accumulation of key heavy metals in grains, such as Arsenic (As), Chromium (Cr), Mercury (Hg), Lead (Pb), and Zinc (Zn), exhibited no significant differences between the treatment and non-fertilized control (see **Table 4**). However, prolonged use of mineral fertilization alone led to higher concentrations of Cadmium (Cd), Nickel (Ni), and Copper (Cu) in grains compared to the non-fertilised control group. Pig slurry (PS) and chicken manure (CM) treatments fell in between, with concentrations that did not significantly differ from either mineral fertilization (Cd) or the control groups (Cd, Ni, Cu).

**Table 4:** Mean concentration of principal heavy metals found in rice grains in 2021 after five years of four fertilization strategies.

	Mean concentration (mg kg^-1^)							
Fertilization	As	Cd	Cr	Ni	Hg	Pb	Cu	Zn
	ns	*	ns	**	ns	ns	***	ns
MIN	0.57 a	0.013 a	0.025 a	0.42 a	0 a	0.27 a	3.20 a	20.50 a
PS	0.68 a	0.010 ab	0.023 a	0.33 b	0 a	0.22 a	2.83 b	20.98 a
CM	0.69 a	0.009 ab	0.005 a	0.33 b	0 a	0.29 a	2.73 b	19.83 a
C	0.66 a	0.007 b	0.028 a	0.31 b	0 a	0.28 a	2.70 b	20.00 a

MIN, mineral fertilizer; PS, pig slurry; CM, chicken manure; C, control. Measurements are shown as means. Different lowercase letters indicate significant differences at p<0.05 between treatments (one-way ANOVA), as determined using Tukey’s test (HSD). ns, no significance, *p<0.05, **p<0.01, *** p<0.001 according to one-way (fertilization) ANOVA test.

### 3.2 NDVI measured with handheld sensor and satellite images from Sentinel-2 as a remote tool to detect crop development changes in rice due to different organic and mineral fertilization strategies

**Figure 4:**
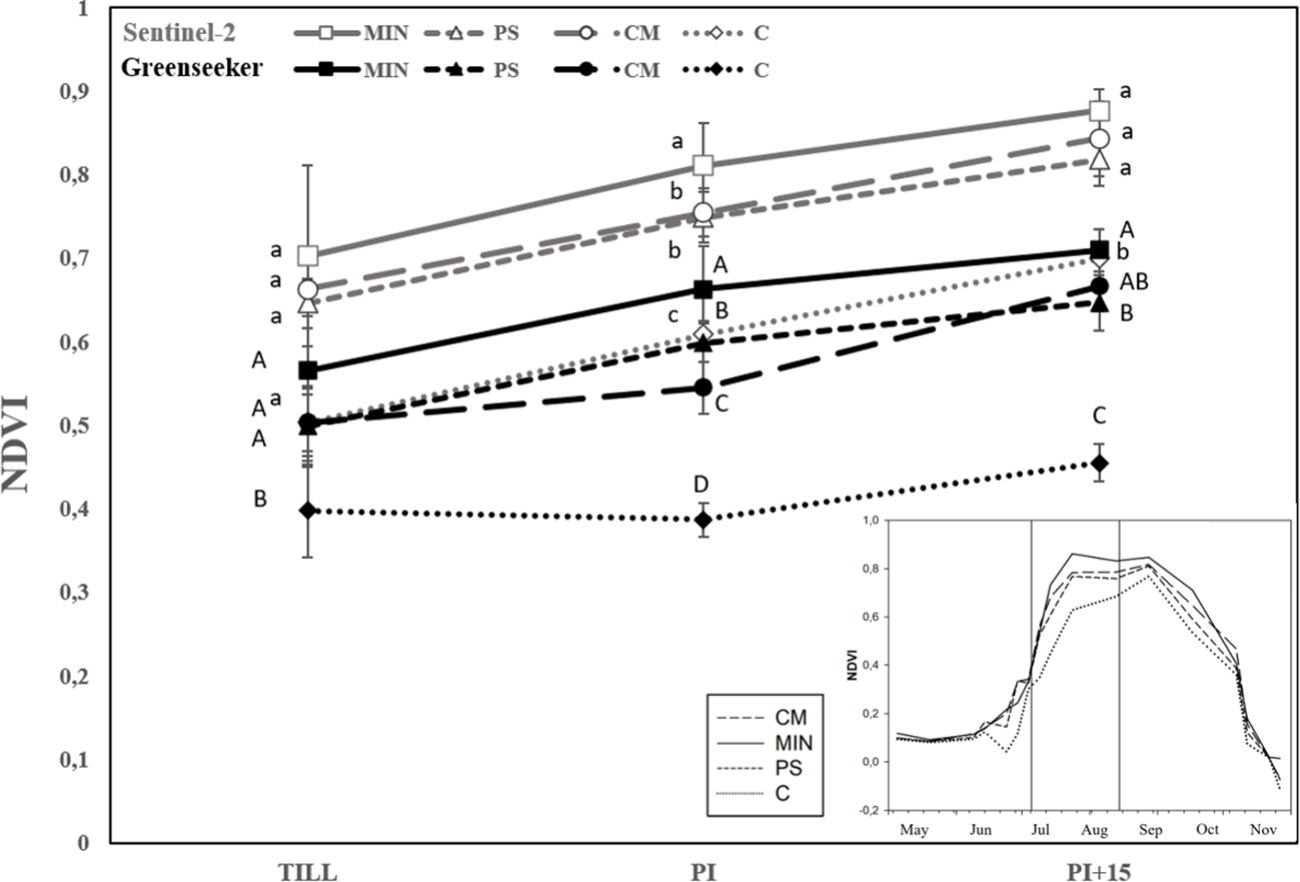
NDVI evolution during crop development measured at ground level by the GreenSeeker and satellite-derived from Sentinel-2 for each N treatment. Means of the six-year trial are represented in the enlarged graph. NDVI evolution of 2021 is shown in the bottom right corner graph. MIN, mineral fertilizer; PS, pig slurry; CM, chicken manure; C, control. Till = maximum tillering stage of rice, PI = panicle initiation stage, PI+15 = 15 days after panicle initiation. Different lowercase letters indicate significant differences at *p*<0.05 between treatments in NDVI Sentinel-2, as determined using Tukey’s test (HSD). Different uppercase letters indicate significant differences at *p*<0.05 between treatments in NDVI Greenseeker, as determined using Tukey’s test (HSD). Measurements are shown as mean ± Standard Error (SE). Standard error is shown as error bars.

**Figure 5:**
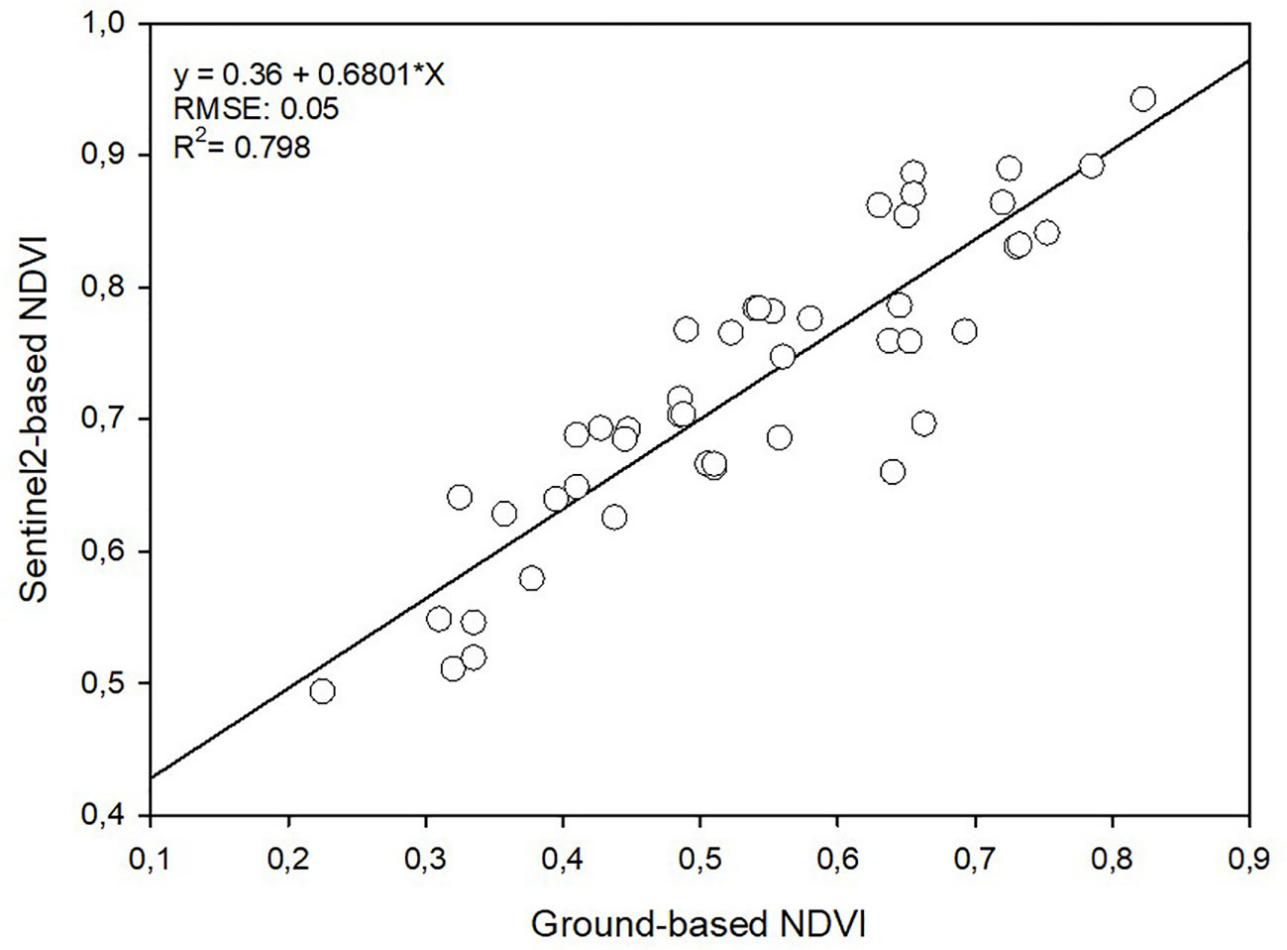
Linear regression between ground-based and satellite-based (Sentinel-2) NDVI.

The NDVI measured using a handheld optical sensor called GreenSeeker at three different growth stages (tillering, panicle initiation, and 15 days after panicle initiation), demonstrated the following trends: 1) in the MIN treatment, it was 55% higher at tillering, 45% higher at panicle initiation, and 47% higher at 15 days after panicle initiation compared to the control (see **Figure 4**); 2) In the PS treatment, NDVI values were 31% higher at tillering, 26% higher at panicle initiation, and 38% higher at 15 days after panicle initiation compared to the control; 3) In the CM treatment, NDVI values were 31% higher at tillering, 26% higher at panicle initiation, and 34% higher at 15 days after panicle initiation compared to the control. Furthermore, it’s worth highlighting that NDVI values extracted from satellite images using Sentinel-2 consistently exceeded the ground-level values obtained with GreenSeeker by 26% (as depicted in **Figure 4**). Despite this difference in magnitude, it is essential to underscore the significant and positive correlation observed between the ground-based and satellite-based NDVI measurements. This correlation is particularly noteworthy, with a coefficient of determination (R^2^) value of 0.798, as illustrated in **Figure 5**.

**Figure 6:**
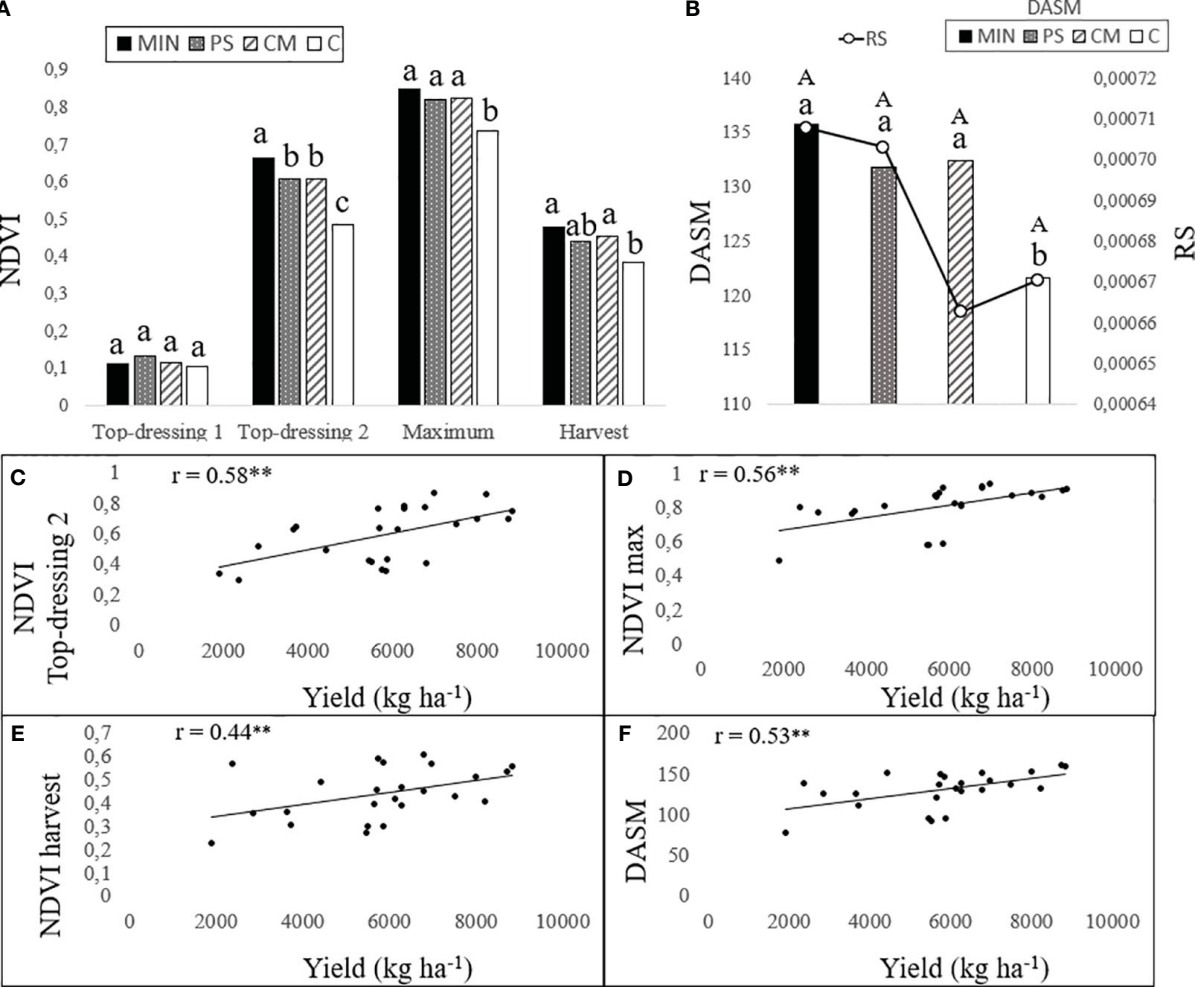
NDVI Sentinel-2 derived data in organic and mineral fertilization strategies and correlations with yield: **(A)** Six-year satellite derived maximum NDVI and NDVI mean at three crop developmental stages: 3-4 leaves stage, panicle initiation (top dressing 1 and 2 respectively), and before harvesting; **(B)** Days to maturity (DASM) and rate of senescence (RS); **(C)** Linear correlation analysis between NDVI at top-dressing 2 and yield; **(D)** Linear correlation analysis between maximum NDVI and yield; **(E)** Linear correlation analysis between NDVI at harvest and yield; **(F)** Linear correlation analysis between DASM and yield. MIN, mineral fertilizer; PS, pig slurry; CM, chicken manure; C, control. NDVI Top-dressing 1 = NDVI at first top-dressing application, NDVI Top-dressing 2 = NDVI at second top-dressing application, NDVImax = maximum NDVI value during crop development, NDVI before harvesting, DASM = days after sowing to physiological maturity, RS = rate of senescence. Measurements are shown as means. Different lowercase letters indicate significant differences in Top-dressing 1, Top-dressing 2, Maximum, Harvest and DASM at *p<0.05* between treatments (one-way ANOVA), as determined using Tukey’s test (HSD). Different uppercase letters indicate significant differences in RS at *p<0.05* between treatments (one-way ANOVA), as determined using Tukey’s test (HSD). Significance of Pearson correlation coefficient (r) is shown as, **p<0.01, according to one-way (fertilization) ANOVA test. Linear relationships between the studied variables and yield were evaluated by the Pearson correlation coefficient (r) (*p*<0.05), only significant correlations are illustrated.

The evaluation of NDVI through Sentinel-2 during crop development provided insights into the performance of plants under different fertilization treatments. Notably, plants under PS and CM exhibited growth limitations between the first and second top-dressing applications, resulting in a lower growth capacity during these developmental phases compared to the MIN treatment (see **Figure 6**). This trend aligns with the observations made using ground based NDVI measurements. However, it is important to highlight that the organic fertilization treatments with PS and CM were able to recover and maintain NDVI values equivalent to the MIN treatment once the second topdressing was applied.

**Figure 7:**
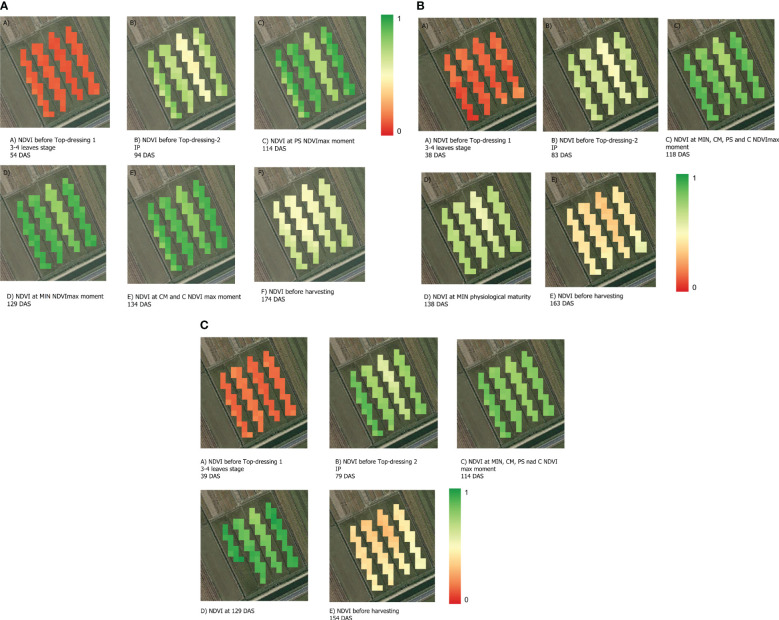
Spectral response (NDVI) of rice crops to four different N fertilization strategies (MIN, PS, CM and C) and their evolution throughout crop development in three representative years of the trial. MIN, mineral, PS, pig slurry; CM, chicken manure; C, control; PI, panicle initiation stage; DAS, days after sowing. Only central pixels that were entirely inside each plot were considered. Representative years of the trial are presented in **(A)** 2018, **(B)** 2020, and **(C)** 2021.

The satellite image data collected throughout the rice crop cycle showed similar trends in the spectral response to N fertilization strategies across years. Three representative years of the study are presented in **Figure 7**. The years 2018, 2020 and 2021 were selected as representative due to the large availability of optimal satellite images (without clouds) throughout the crop cycle, and to less crop affectation of uncontrollable factors alien to N fertilization compared to the other years. The NDVI values were similar among the four strategies in the early stages of plant development, with low NDVI values being associated with low plant coverage at the 3–4 leaf physiological stage. However, later in the crop cycle, different spectral developments were observed between fertilization strategies (**Figures 4**, **7**). The MIN strategy reached the highest NDVI value at the panicle initiation stage, followed by PS- and CM-fertilised plants and the C plants showed a much lower NDVI value. The MIN, CM, and PS strategies achieved similar maximum NDVI values, every year approximately twenty days after the second top-dressing at panicle initiation. In general, the plants in strategy C reached physiological maturity earlier than the other three fertilization strategies and MIN and CM had higher NDVI values at harvest than C. Finally, the rate of senescence was similar in all fertilization treatments (**Figure 6**).

### 3.3 Long-term (6 years) effects of organic and mineral fertilization strategies on soil nutrient contents in a rice field crop

The initial soil nutrient contents available at the 2^nd^ year of the experiment (2018) was similar among all experimental plots in terms of soil moisture, organic carbon, total N, available P, and available K (**Table 5**). However, after six years of the application of four fertilization strategies, soils accumulated different moisture, P and K contents (**Table 5**). Six-year CM fertilization significantly enriched the soil in both total (16% MIN, 20% PS, 23% C) and available (86% MIN, 49% PS, 42%C) P and available K (20% MIN, 13%PS, 21%C) with respect to the rest of strategies due to its accumulation over the years. Six-year MIN fertilization impoverished the soil available P by 24% compared to the control (zero fertilized strategy). Soil moisture was maintained by the continuous application of CM, whereas it was reduced by the continuous application of the other strategies. Soil total nitrogen content was similar in all fertilization strategies at the beginning and end of the six-year experiment.

**Table 5:** Soil properties at the beginning of the trial (2^nd^ year) and after six-year rice cultivation with fertilization using different N fertilization strategies.

Year	Fertilization	Soil Moisture (%)	Soil organic carbon (%)	Total soil N (% s.m.s.)	Total soil P (mg kg^-1^)	Soil available P (mg kg^-1^)	Total soil K (mg kg^-1^)	Soil available K (mg kg^-1^)
		ns	ns	ns	ns	ns	ns	ns
2018*	MIN	22.0 a	3.5 a	0.207 a	737.0 a	18.52 a	5733.7 a	154.7 a
	PS	22.4 a	3.5 a	0.209 a	711.75 a	18.47 a	5536.5 a	144.0 a
	CM	21.5 a	3.5 a	0.208 a	762.5 a	18.12 a	7104.5 a	147.7 a
	C	21.9 a	3.5 a	0.205 a	719.5 a	17.12 a	4885.5 a	143.2 a
		**	ns	ns	***	***	ns	***
2022	MIN	20.4 b	3.0 a	0.212 a	743.75 b	15.97 c	8723.5 a	153.2 b
	PS	20.1 bc	3.1 a	0.207 a	721.25 b	20.05 bc	8749.25 a	162.0 b
	CM	21.1 a	3.1 a	0.215 a	862.25 a	29.78 a	8908.75 a	183.2 a
	C	19.6 c	2.9 a	0.192 a	702.75 b	20.95 b	7643.25 a	151.2 b
	Sources	p(F)						
	Fertilization	0.054	0.147	0.03	0.0002	<0.0001	0.106	0.0003
	Year	<0.0001	<0.0001	0.87	0.1008	<0.0001	<0.0001	<0.0001
	Fertilization × Year	<0.001	0.575	0.114	0.0464	<0.0001	0.73	<0.001

MIN, mineral fertilizer; PS, pig slurry; CM, chicken manure; C, control. Measurements are shown as means. Different lowercase letters indicate significant differences at p<0.05 between treatments (one-way ANOVA), as determined using Tukey’s test (HSD). ns, no significance, **p<0.01, *** p<0.001 according to one-way (fertilization) ANOVA test.* Note that soil samples were taken for macronutrients only in the 2^nd^ year of the experiment and samples were not available for 2017.

Changes in soil water nitrate and ammonium concentrations throughout the crop cycle showed similar trends for the different fertilization strategies across the years (**Supplementary Figure 1**). In general, the levels of both forms of N were lower at 60 cm than at 30 cm. Nitrate and ammonium peaks always appeared within a few days of flooding at both depths (**Supplementary Figure 1**). Regarding nitrates in the plant-usable soil water fraction (-30 cm), the peak was generally higher with MIN fertilization. The MIN strategy maintained higher nitrate concentrations from the beginning of the field flooding. In addition, it maintained higher nitrate concentrations for longer periods, generally having a delayed decline compared with the other strategies (**Supplementary Figure 1**). MIN showed the highest peak of ammonium in soil water after field flooding. However, PS showed a higher supply of available ammonium than CM. The contribution of ammonium in CM was always very low. Ammonium sulphate (second topdressing) at panicle initiation (PI) did not produce a peak of nitrate or ammonium at either depth. As for the 60 cm depth, MIN provided the highest nitrate and ammonium content, although in some years it increased in the organic strategies.

**Figure 8:**
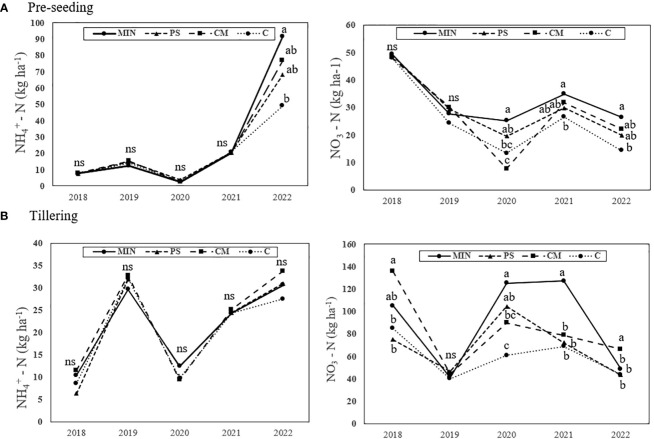
Long-term progressions of nitrate and ammonium soil concentrations measured at two different times: before sowing and before the first top-dressing application (tillering). MIN, mineral fertilizer; PS, pig slurry; CM, chicken manure; C, control. NH_4_^+^ – N, ammonium; NO_3_^-^ N, nitrate. No ammonium data were available for PS 2020 during the tillering stage. Different letters indicate significant differences at *p*<0.05, as determined using Tukey’s test (HSD). ns, non significant.

There were no differences in soil ammonium concentrations between the strategies over time before the first topdressing was applied (**Figure 8**). In contrast, soil nitrate content varied throughout the study period and generally, the MIN and CM strategies had greater soil nitrate content, which was an expected result considering that they are the only two strategies with basal fertilizer application.

**Figure 9:**
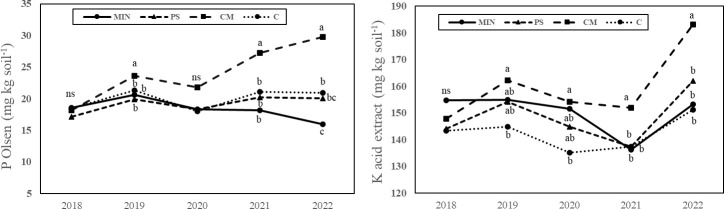
Long-term progressions of available phosphorus (P) and potassium (K) measured before sowing each year. MIN, mineral fertilizer; PS, pig slurry; CM, chicken manure; C, control. Different letters indicate significant differences at p<0.05, as determined using Tukey’s test (HSD). ns, non significant.

Six-year CM application resulted in an increase of 64% and 24% of soil available P and K content as compared to C treatment (**Figure 9**). However, the long-term stand-alone mineral nitrogen fertilizer application did not affect available K content but decreased available P content by 14% compared to no-fertilization, although it was supplemented with calcium superphosphate as no fertilised plot. Long-term PS application showed similar P and K contents to the control (**Figure 9**).

## 4 Discussion

### 4.1 Rice yields and yield components negatively impacted by organic as compared to mineral fertilization with long term grain heavy metals accumulation within safe contents

Nitrogen fertilization has a crucial impact on rice crops. Previous studies have suggested that the nutrient form, source rate, timing of application and the ecosystem in which it is used are essential factors that modify Nitrogen Use Eefficiency (NUE), crop development and yield (Geng et al., 2019; Didal et al., 2022). All parameters contributing to the yield and its components are affected by N supply, including vegetative biomass, tillering capacity, panicle number, and percentage of filled grains, as N is an essential constituent of amino acids, nucleic acids, and chlorophyll (Qiao et al., 2013; Padhan et al., 2023). Consequently, leaf N concentration is closely related to the photosynthesis rate, crop biomass production, and grain yield (Dobermann and Fairhurst, 2000; Ding et al., 2014; Duque et al., 2023).

During the early stages of plant growth (germination and seedling growth), rice takes up a small amount of N; therefore, differences in fertilization management were not visible. As the root system, leaves and stems develop, the plant increases N uptake, reaching a maximum between the tillering and flowering stages (Linquist et al., 2006; Rehman et al., 2023). Once the root system develops into many superficial roots, N uptake by the plant is high (Dobermann and Fairhurst, 2000; Rehman et al., 2023). Finally, during the reproductive phase, N uptake ceases owing to the remobilization and reallocation of N from vegetative tissues to reproductive organs (Geisseler and Horwath, 2018). At maturity, more than half of the aboveground N in the plant is found in the grains (Linquist et al., 2006; Somaweera et al., 2016).

In the present study, only the zero-N fertilised plants showed a lower tillering capacity, and both animal manure strategies (CM and PS) developed a tillering capacity similar to that of mineral-fertilised plants (MIN). It is well known that the application of N fertilizer increases the cytokine content within tiller nodes and further enhances the tillering capacity (Liu et al., 2011; Wang et al., 2017; Zha et al., 2022), suggesting that all three strategies applied in this study supplied a similar amount of nitrogen at the vegetative phase. However, a clear decrease in panicle density was reported in CM and PS compared to MIN, which may suggest that there is a limitation in N supply during the tillering to panicle initiation period. Although rice requires N throughout its growth period, the greatest requirements occur between the early and mid-tillering and panicle initiation stages (Dobermann and Fairhurst, 2000; Bashir et al., 2023). At these critical growth stages, there is a large N demand and rapid biomass accumulation when nutritional deficiencies usually appear, which is consistent with the results of our study regarding the effects of different organic fertilizer strategies. Growth limitations owing to N deficiency were also demonstrated by a reduction in height and accelerated senescence in the CM and PS strategies.

Important concerns have been raised regarding the safety of organic fertilizers for food production (Alam et al., 2020). Heavy metals can be toxic to humans and plants at low concentrations and can cause nutrient imbalances (Czarnecki and Düring, 2015; Zhao and Wang, 2020), hence the long-term heavy metal accumulations were also assessed in this study. Several studies have reported that the long-term application of animal manure (such as pig and chicken manure) can cause soil pollution and heavy metal grain accumulation as many farmland manures are contaminated with heavy metals, thus posing an unacceptable risk to the environment and human health (Wang et al., 2016; Lan et al., 2022). The results presented herein showed an increased accumulation of Ni and Cu in the grains of the MIN-treated plots. Previous studies have demonstrated that heavy metal uptake and accumulation by plants depend on their availability in the soil (Xiao et al., 2011; Duan et al., 2022). It has been described that there are multiple factors influencing heavy metal bioavailability, the soil pH being the main one (Wei et al., 2020). Nitrate and ammonium ions are taken up from the medium through different mechanisms by which chemical changes are generated in the surrounding rhizosphere (Begum et al., 2023), thus generating differences in the bioavailability of heavy metals in the soil. The form of N fertilizer can affect the bioavailability of heavy metals and thus their accumulation in grains (Xiao et al., 2011; Begum et al., 2023).

A six-year application of animal manure for fertilization did not influence the accumulation of analysed heavy metals in grains when compared to the control (C). This implies that there is no additional risk to consumer health associated with these manure-based fertilization strategies, and in certain cases, they even led to a reduction in grain heavy metal content compared to exclusive mineral fertilization. Our results suggest that the strategies proposed in this study do not affect grain heavy metal uptake and accumulation, however it is always advisable to monitor manures before their field application to avoid possible associated risks (Qaswar et al., 2020; Wan et al., 2020).

### 4.2 Proximal and satellite-based Sentinel-2 NDVI measurements in rice fields effectively identify nutrient deficiencies in organic fertilization strategies

During critical rice developmental stages like tillering and panicle initiation, timely nutrient management is essential to ensure that topdressing N applications match crop requirements and prevent growth limitations. In our study, after the second topdressing at panicle initiation with ammonium sulphate, rice crops fertilized with CM and PS demonstrated remarkable recovery, as evidenced by the significant increase in NDVI values, which closely approached those of the mineral fertilization strategy. The supplementation of ammonium sulphate effectively facilitated proper panicle development and grain filling, resulting in highly productive panicles comparable to those in the MIN strategy. Maximum NDVI values were attained within a few days of panicle initiation, with no significant differences observed among the MIN, CM, and PS strategies. However, during the period between tillering and panicle initiation, rice plots fertilized with organic amendments exhibited lower NDVI values. This period corresponded to a phase when nitrogen deficiency negatively impacted the crop, leading to reduced panicle numbers and yields when using PS or CM. This observation underscores the efficacy of mineral topdressing in complementing organic fertilization methods, enabling plants to reach similar vigour at maturity with similar senescence rates and crop cycles. The decline in NDVI before panicle development accurately reflected nutritional deficiencies, which can be detected through NDVI readings. This suggested that real-time monitoring of rice crops using NDVI measurements can aid in determining the ideal timing for topdressing in organic treatments. Detecting a decline in NDVI early on, as a basis for topdressing recommendations, can prevent yield losses associated with suboptimal resource management. Therefore, the results highlight the significant potential of Sentinel-2 image analysis for the early detection of nutritional stress in rice crops. The reliability of satellite-derived vegetation indices was substantiated by their strong correlation with ground-based handheld sensor readings. Other researchers have also validated NDVI Sentinel-2 with proximal measurements (Tittebrand et al., 2009; Mzid et al., 2020; Segarra et al., 2020). In conclusion, the suitability of Sentinel-2 as a remote sensing tool for crop monitoring is well-established. Moreover, the implementation of remote sensing with Sentinel-2 should be considered as a straightforward and readily available approach for the early detection of nutritional deficiencies resulting from organic fertilization and for monitoring mineral fertilizer supplementation in accordance with crop requirements. This tool can facilitate the adjustment of fertilization schedules in real time, offering a fast and precise means to ensure high productivity and efficient resource utilization. Furthermore, it holds promise for future crop monitoring endeavours, providing data that is comparable to handheld sensors while offering the advantages of large-scale application, resulting in significant savings in resources and time.

While the issue of NDVI saturation has been widely documented in other studies and overcome by the application of alternative vegetation indices (Rehman et al., 2019; Jiang et al., 2021; San Bautista et al., 2022), our research did not encounter this limitation due to the low-density cultivation conditions, low yields (below 8,000 kg ha^-1^), and sparse biomass coverage (considering the crop was dry seeded).

### 4.3 Durable soil nutrient dynamics of mineral fertilization strategies provide increased N availability to rice crops as compared to organic fertilizer strategies

The Agronomic Efficiency Associated with Total Nitrogen for the CM and PS strategies was lower than that for the MIN strategy, primarily due to differences in the nitrogenous forms present in these fertilizers. In the MIN strategy, the agronomic efficiency associated with total nitrogen was 22.2 grain Kg/Kg N, which exceeded the values observed in both animal manure strategies but fell within the optimal range established in previous studies (Ladha et al., 2005; Xie et al., 2007; Moreno-García et al., 2017). Notably, there was no significant difference in agronomic efficiency associated with ammonium among the three strategies. This result aligns with the findings of Moreno-García et al. (2017), who concluded that rice crops primarily utilize inorganic N in the short term, with limited uptake of organic N. Both CM and PS contain a higher proportion of organic nitrogen (approximately 42% and 34%, respectively) compared to mineral forms, with a slightly higher percentage of ammonium nitrogen in PS than in CM. In summary, the application of organic fertilizers in rice cultivation can provide long-term nutrient benefits when properly managed in agricultural practices. However, this approach comes with trade-offs, including slower nutrient release and the potential for early growth limitations if not properly managed. Various pre-treatment techniques, such as mineralization, acidification, and digestion, have been developed to convert nitrogenous forms and organic compounds into more readily accessible N formulations for plants (Bosshard et al., 2010). Improved NUE could also contribute to reduced pollutant emissions. However, the practical benefits of pre-treated animal slurry in terms of N availability for NUE enhancement have shown variability under field conditions, as reported by previous studies (Moeller and Mueller, 2012). Additionally, it is crucial that such treatments do not substantially increase fertilizer costs, as is often the case with current methods requiring expensive equipment and significant energy demands (Dadrasnia et al., 2021). Further research into animal manure pre-treatment technologies is warranted, as they hold the potential to enhance N efficiency in manure while simultaneously reducing N emissions into the environment.

Soil nitrate-N levels before applying any amendment vary annually due to their sensitivity to rainfall and environmental conditions (Liang et al., 1991; Zhao et al., 2023). During the period from sowing to flooding, nitrate N undergoes denitrification and quickly releases as N_2_ and N_2_O following flooding (Ponnamperuma, 1972; Li et al., 2018). In contrast, ammonium N, produced through N mineralization under these flooded conditions, tends to accumulate (Dobermann and Fairhurst, 2000; Alam et al., 2019). Consequently, to prevent nitrification and subsequent denitrification, it is advisable to apply basal N fertilizers as close to flooding as possible. In this study, rice was sown well in advance of flooding, a period spanning 38 days. During this time, nitrate generated through mineralization processes persisted in the soil, making it accessible to the crop during the initial stages of plant growth. Additionally, in the MIN strategy, the soil was enriched with urea, while in the CM strategy, manure was incorporated into the soil. These practices effectively curtailed surface ammonia volatilization (Mutters et al., 2010; Gaihre et al., 2023). Previous studies show the NUE improvement that involves the application of N fertilizers in multiple split doses (Li et al., 2018; Phongchanmixay et al., 2019; Jiang-ming, 2023). However, many split fertilizations also increase farmer’s labour output and crop damage, reducing net benefits. In addition, there is no machinery available to apply the animal manures used once the field is flooded, which hinders the fractionation of organic fertilization. Therefore, a more efficient schedule must be reconsidered based on plant nutrient status, crop demand and fertilizer composition, to improve yield and reduce N loss.

Ammonium-N is the dominant and preferred form for rice because it requires less energy for metabolism compared to nitrate, although rice can still utilize nitrate. The majority of absorbed ammonium is integrated into organic compounds within the roots. Conversely, nitrate-N is more mobile within the xylem, necessitates conversion to ammonium through nitrate and nitrite reductases, and is stored in the vacuoles (Dobermann and Fairhurst, 2000; Mahboob et al., 2023). Interestingly our study has shown that during flooding in all years, both the MIN and CM strategies exhibited significant peaks of water nitrate at depths of 30 and 60 cm. This likely resulted from nitrate produced through mineralization occurring between the basal fertilization and flooding stages. Much of this nitrate is susceptible to leaching, with only a small fraction being potentially taken up by plants (Qiu et al., 2022). Meanwhile, the MIN and PS strategies showed an ammonium peak during flooding. Ammonium is less prone to leaching due to its positive charge. Additionally, ammonia losses through volatilization can be substantial, reaching up to 60% of the applied nitrogen. This is because ammonium-N is loosely bound to water molecules and can convert into non-ionized ammonia (NH_3_), which can escape from water in gaseous form (Choudhury and Kennedy, 2005; Gu and Yang, 2022).

The levels of detected ammonium were considerably lower in the MIN strategy, likely due to oxide nitrate-N reduced soil mobility, as it readily adsorbs to clay minerals, is subject to loss through volatilization, and is preferentially taken up by rice plants (Giehl and von Wirén, 2014; Gu and Yang, 2022). In contrast, CM did not provide the same quantity of available ammonium in the soil, resulting in a reduced agronomic efficiency associated with total N for this fertilizer. Root system architecture varies greatly between cultivated rice lines (Uga et al., 2013; Verma et al., 2022). Although it has been reported that root length can reach 50 cm (Uga et al., 2009; Kawai et al., 2022; FAO, 2023), previous studies have suggested that the upper layer (0 -20 cm) of rice roots plays a key role in absorbing moisture and nutrients, which can increase the grain filling rate and grain weight (Deng et al., 2020). Thus, higher concentrations of nitrate below the effective rooting zone in the MIN strategy than in the other strategies suggested that nitrate leaching poses a risk of groundwater pollution (Huang et al., 2017; Amin et al., 2021). CM fertilization can also pose a risk of pollution, although it has a lower impact than the MIN strategy. Moreover, leaching reduced the NUE of the applied fertilizers. However, the PS strategy showed the lowest N loss, owing to leaching together with C. This can be explained by the fact that PS was not used as a basal fertilizer. In contrast, there was a greater rate of ammonium volatilisation in the PS strategy, which could explain why both animal manure strategies showed similar agronomic efficiency.

The utilization of organic amendments in agricultural practices has been proposed by previous research as beneficial for both short-term micro- and macronutrient assimilation and the long-term enhancement of soil quality (Nishikawa et al., 2014; Schmidt and Knoblauch, 2020; Li et al., 2022). However, adequate management of phosphorus and potassium fertilization is essential for sustaining a minimum available supply of these nutrients without restricting plant growth and, consequently, NUE (Dobermann and Fairhurst, 2000; Su et al., 2023; Thanh et al., 2023). The results presented herein demonstrated that prolonged CM application enriched the soil with phosphorus and potassium. In contrast, the MIN strategy led to soil P depletion, potentially attributed to higher extraction by the crop, despite the application of basal P fertilizer to avoid growth limitations. The PS strategy maintained soil P and K levels equivalent to those of the control strategy. Therefore, it would be advantageous to explore various combinations of organic and inorganic fertilizers, employing organic fertilization and supplementing nitrogen deficiencies with mineral fertilizers using real-time remote sensing. Several studies have already demonstrated the potential viability of such resource optimization strategies (Iqbal et al., 2020; Zhang et al., 2020).

## 5 Conclusions

This research provided valuable insights into rice cultivation and fertilization, resulting in practical recommendations for optimizing yield, ensuring food safety, and managing nutrients effectively through both organic and mineral fertilization methods. The study also explored the use of real-time remote sensing, demonstrated by Sentinel-2 NDVI measurements, to enhance precise nutrient management. It emphasized considering manure sources and soil properties with pre-application monitoring. Remote sensing measurements, proved adequate in monitoring nutrient deficiencies and timing of nitrogen application was crucial particularly between the vegetative and panicle initiation stages. The research leaned towards the adoption of cost-effective organic fertilizers, particularly emphasizing the pig slurry strategy, which exhibited the lowest nitrogen loss through leaching. However, it’s important to acknowledge that organic fertilizer applications presented trade-offs, decreasing rice yields compared to mineral fertilization. Safety concerns on heavy metal contamination were addressed, showing no significant increase in rice grains in the organic strategies.

## Data availability statement

The original contributions presented in the study are included in the article/**Supplementary Material**. Further inquiries can be directed to the corresponding author.

## Author contributions

MC-F, CO, and GM conceived the study. MC-F, NT, AV, and KM-J collected data and organised the database. MS-T provided the basic data for the study. AV and KM-J performed the statistical analyses. MC-F, KM-J, and ML contributed to data interpretation. KM-J wrote the first draft of the manuscript. MSL designed the research and revised the manuscript. Each of the authors participated in the manuscript’s revision process and has thoroughly reviewed and given their approval for its submission.

## References

[B1] Alam M. Hussain Z. Khan A. Khan M. A. Rab A. Asif M.. (2020). The effects of organic amendments on heavy metals bioavailability in mine impacted soil and associated human health risk. Scientia Hortic. 262, 109067. doi: 10.1016/j.scienta.2019.109067

[B2] Alam M. Rahman M. Biswas J. C. Akhter S. Maniruzzaman M. Choudhury A. K.. (2019). Nitrogen transformation and carbon sequestration in wetland paddy field of Bangladesh. Paddy Water Environ. 17, 677–688. doi: 10.1007/s10333-019-00693-7

[B3] Amanullah Khan S.-T. Iqbal A. Fahad S. (2016). Growth and productivity response of hybrid rice to application of animal manures, plant residues and phosphorus. Front. Plant Sci. 7. doi: 10.3389/fpls.2016.01440 PMC5067482 27803701

[B4] Amin M. G. M. Akter A. Jahangir M. M. R. Ahmed T. (2021). Leaching and runoff potential of nutrient and water losses in rice field as affected by alternate wetting and drying irrigation. J. Environ. Manage. 297, 113402. doi: 10.1016/j.jenvman.2021.11340 34333312

[B5] Anisuzzaman M. Rafii M. Y. Jaafar N. M. Ramlee S. I. Ikbal M. F. Haque M. A. (2021). Effect of organic and inorganic fertilizer on the growth and yield components of traditional and improved rice (Oryza sativa l.) genotypes in Malaysia. Agronomy 11 (9), 1830. doi: 10.3390/agronomy11091830. Article 9.

[B6] Bashir S. S. Siddiqi T. O. Kumar D. Ahmad A. (2023). Physio-biochemical, agronomical, and gene expression analysis reveals different responsive approach to low nitrogen in contrasting rice cultivars for nitrogen use efficiency. Mol. Biol. Rep. 50 (2), 1575–1593. doi: 10.1007/s11033-022-08160-z 36520360

[B7] Begum M. Li L. Young E. Carey M. Li G. Zhu Y.-G.. (2023). Fertilization enhances grain inorganic arsenic assimilation in rice. Exposure Health. doi: 10.1007/s12403-023-00563-y

[B8] Bosshard C. Flisch R. Mayer J. Basler S. Hersener J. L. Meier U.. (2010). Improving nitrogen efficiency *via* slurry treatment. Recherche Agronomique Suisse (10), 378–383. Available at: https://www.cabdirect.org/cabdirect/abstract/20103336107.

[B9] Brunelle T. Dumas P. Souty F. Dorin B. Nadaud F. (2015). Evaluating the impact of rising fertilizer prices on crop yields. Agric. Economics 46 (5), 653–666. doi: 10.1111/agec.12161

[B10] Cassman K. G. Peng S. Olk D. C. Ladha J. K. Reichardt W. Dobermann A.. (1998). Opportunities for increased nitrogen-use efficiency from improved resource management in irrigated rice systems. Field Crops Res. 56 (1), 7–39. doi: 10.1016/S0378-4290(97)00140-8

[B11] Chauhan B. S. Jabran K. Mahajan G. (2017). Rice Production Worldwide (Germany: Springer International Publishing). doi: 10.1007/978-3-319-47516-5

[B12] Choudhury A. T. M. A. Kennedy I. R. (2005). Nitrogen fertilizer losses from rice soils and control of environmental pollution problems. Commun. Soil Sci. Plant Anal. 36 (11–12), 1625–1639. doi: 10.1081/CSS-200059104

[B13] Czarnecki S. Düring R.-A. (2015). Influence of long-term mineral fertilization on metal contents and properties of soil samples taken from different locations in Hesse, Germany. Soil 1 (1), 23–33. doi: 10.5194/soil-1-23-2015

[B14] Dadrasnia A. de Bona Muñoz I. Yáñez E. H. Lamkaddam I. U. Mora M. Ponsá S.. (2021). Sustainable nutrient recovery from animal manure: A review of current best practice technology and the potential for freeze concentration. J. Cleaner Production 315, 128106. doi: 10.1016/j.jclepro.2021.128106

[B15] Deng J. Feng X. Wang D. Lu J. Chong H. Shang C.. (2020). Root morphological traits and distribution in direct-seeded rice under dense planting with reduced nitrogen. PloS One 15 (9), e0238362. doi: 10.1371/journal.pone.0238362 32877452 PMC7467324

[B16] Didal V. Vidyasagar G. Mahender Kumar R. Surekha K. Narender Reddy S. Bhooshan B. (2022). Effect of nitrogen management practices on SPAD values and NDVI readings of rice crop. Pharma Innovation J. 11 (2), 367–371. Available at: https://www.thepharmajournal.com/archives/2022/vol11issue2/PartF/11-1-399-395.pdf.

[B17] Ding W. Xu X. He P. Ullah S. Zhang J. Cui Z.. (2018). Improving yield and nitrogen use efficiency through alternative fertilization options for rice in China: A meta-analysis. Field Crops Res. 227, 11–18. doi: 10.1016/j.fcr.2018.08.001

[B18] Ding C. You J. Chen L. Wang S. Ding Y. (2014). Nitrogen fertilizer increases spikelet number per panicle by enhancing cytokinin synthesis in rice. Plant Cell Rep. 33 (2), 363–371. doi: 10.1007/s00299-013-1536-9 24258242

[B19] Dobermann A. Fairhurst T. (2000). Rice: Nutrient Disorders & Nutrient Management. Available at: https://api.semanticscholar.org/CorpusID:126596584.

[B20] Duan Y. Li Q. Zhang L. Huang Z. Zhao Z. Zhao H.. (2022). Toxic metals in a paddy field system: A review. Toxics 10 (5), 249. doi: 10.3390/toxics10050249 35622662 PMC9148070

[B21] Duque A. F. Patino D. Colorado J. D. Petro E. Rebolledo M. C. Mondragon I. F.. (2023). Characterization of rice yield based on biomass and SPAD-based leaf nitrogen for large genotype plots. Sensors 23 (13), 5917. doi: 10.3390/s23135917. Article 13. 37447767 PMC10347115

[B22] ESA. (2023). Sentinel-2 Mission Guide. Available at: https://sentinel.esa.int/web/sentinel/missions/sentinel-2.

[B23] EUROSTAT. (2023). Pig Population. Available at: https://ec.europa.eu/eurostat/databrowser/view/apro_mt_lspig/default/table?lang=en.

[B24] FAO. (2023). Scaling soil nutrient balances. Available at: https://www.fao.org/3/y5749e/y5749e0j.htm#TopOfPage.

[B25] FAOSTAT. (2021). Crops and livestock products. Available at: https://www.fao.org/faostat/en/#data/QCL/visualize.

[B26] Fixen P. E. Johnston A. M. (2012). World fertilizer nutrient reserves: A view to the future. J. Sci. Food Agric. 92 (5), 1001–1005. doi: 10.1002/jsfa.4532 22415449

[B27] Gaihre Y. K. Bible W. D. Singh U. Sanabria J. Baral K. R. (2023). Mitigation of nitrous oxide emissions from rice-wheat cropping systems with sub-surface application of nitrogen fertilizer and water-saving irrigation. Sustainability 15 (9), 7530. doi: 10.3390/su15097530

[B28] Geisseler D. Horwath W. R. (2018). California Crop Fertilization Guidelines (Davis, CA, USA: UCANR Publication).

[B29] Geisseler D. Scow K. M. (2014). Long-term effects of mineral fertilizers on soil microorganisms – A review. Soil Biol. Biochem. 75, 54–63. doi: 10.1016/j.soilbio.2014.03.023

[B30] Geng Y. Cao G. Wang L. Wang S. (2019). Effects of equal chemical fertilizer substitutions with organic manure on yield, dry matter, and nitrogen uptake of spring maize and soil nitrogen distribution. PloS One 14 (7), e0219512. doi: 10.1371/journal.pone.0219512 31287845 PMC6615609

[B31] Giehl R. F. H. von Wirén N. (2014). Root nutrient foraging. Plant Physiol. 166 (2), 509–517. doi: 10.1104/pp.114.245225 25082891 PMC4213083

[B32] Gu J. Yang J. (2022). Nitrogen (N) transformation in paddy rice field: Its effect on N uptake and relation to improved N management. Crop Environ. 1 (1), 7–14. doi: 10.1016/j.crope.2022.03.003

[B33] Hassan M. A. Yang M. Rasheed A. Yang G. Reynolds M. Xia X.. (2019). A rapid monitoring of NDVI across the wheat growth cycle for grain yield prediction using a multi-spectral UAV platform. Plant Sci. 282, 95–103. doi: 10.1016/j.plantsci.2018.10.022 31003615

[B34] Hirel B. Tétu T. Lea P. J. Dubois F. (2011). Improving nitrogen use efficiency in crops for sustainable agriculture. Sustainability 3 (9), 1452–1485. doi: 10.3390/su3091452. Article 9.

[B35] Hu B. Wang W. Chen J. Liu Y. Chu C. (2022). Genetic improvement toward nitrogen-use efficiency in rice: lessons and perspectives. Mol. Plant 16 (2), 64–74. doi: 10.1016/j.molp.2022.11.007 36380584

[B36] Huang J. Duan Y. Xu M. Zhai L. Zhang X. Wang B.. (2017). Nitrogen mobility, ammonia volatilization, and estimated leaching loss from long-term manure incorporation in red soil. J. Integr. Agric. 16 (9), 2082–2092. doi: 10.1016/S2095-3119(16)61498-3

[B37] Huang L. Yang J. Gao W. Yang W. Cui X. Zhuang H. (2016). Effects of pig slurry as basal and panicle fertilizer on trace element content and grain quality in direct-seeding rice. Sustainability 8 (8), 714. doi: 10.3390/su8080714. Article 8.

[B38] Iqbal A. He L. Ali I. Ullah S. Khan A. Khan A.. (2020). Manure combined with chemical fertilizer increases rice productivity by improving soil health, post-anthesis biomass yield, and nitrogen metabolism. PloS One 15 (10), e0238934. doi: 10.1371/journal.pone.0238934 33027309 PMC7540855

[B39] IRRI. (2002). “International rice research institute,” in Standard evaluation system for rice (Phillipines). Available at: http://www.knowledgebank.irri.org/images/docs/rice-standard-evaluation-system.pdf.

[B40] Jiang R. Sanchez-Azofeifa A. Laakso K. Wang P. Xu Y. Zhou Z.. (2021). UAV-based partially sampling system for rapid NDVI mapping in the evaluation of rice nitrogen use efficiency. J. Cleaner Production 289, 125705. doi: 10.1016/j.jclepro.2020.125705

[B41] Jiang-ming Z. (2023). Improving fertilization practices to reduce the potential of nutrient loss from rice paddy fields. Paddy Water Environ. 21 (1), 115–126. doi: 10.1007/s10333-022-00917-3

[B42] Kawai T. Chen Y. Takahashi H. Inukai Y. Siddique K. H. M. (2022). Rice genotypes express compensatory root growth with altered root distributions in response to root cutting. Front. Plant Sci. 13. doi: 10.3389/fpls.2022.830577 PMC8919052 35295630

[B43] Kjeldahl J. (1883). Neue Methode zur Bestimmung des Stickstoffs in organischen Körpern. Z. Für Analytische Chemie 22 (1), 366–382. doi: 10.1007/BF01338151

[B44] Ladha J. K. Pathak H. Krupnik T. J. Six J. van Kessel C. (2005). Efficiency of fertilizer nitrogen in cereal production: retrospects and prospects. Adv Agron 87, 85–156. doi: 10.1016/S0065-2113(05)87003-8

[B45] Lan W. Yao C. Luo F. Jin Z. Lu S. Li J.. (2022). Effects of application of pig manure on the accumulation of heavy metals in rice. Plants 11 (2), 207. doi: 10.3390/plants11020207. Article 2. 35050095 PMC8777798

[B46] Li X. Li B. Chen L. Liang J. Huang R. Tang X.. (2022). Partial substitution of chemical fertilizer with organic fertilizer over seven years increases yields and restores soil bacterial community diversity in wheat-rice rotation. Eur. J. Agron. 133, 126445. doi: 10.1016/j.eja.2021.126445

[B47] Li G. Lin J. Xue L. Ding Y. Wang S. Yang L. (2018). Fate of basal N under split fertilization in rice with 15N isotope tracer. Pedosphere 28 (1), 135–143. doi: 10.1016/S1002-0160(17)60407-7

[B48] Liang B. C. Remillard M. MacKenzie A. F. (1991). Influence of fertilizer, irrigation, and non-growing season precipitation on soil nitrate-nitrogen under corn. J. Environ. Qual. 20 (1), 123–128. doi: 10.2134/jeq1991.00472425002000010019x

[B49] Linquist B. A. Brouder S. M. Hill J. E. (2006). Winter straw and water management effects on soil nitrogen dynamics in California rice systems. Agron. J. 98 (4), 1050–1059. doi: 10.2134/agronj2005.0350

[B50] Liu Y. Ding Y. Wang Q. Meng D. Wang S. (2011). Effects of nitrogen and 6-benzylaminopurine on rice tiller bud growth and changes in endogenous hormones and nitrogen. Crop Sci. 51 (2), 786–792. doi: 10.2135/cropsci2010.04.0217

[B51] Liu J. Xie Q. shi Q. Li M. (2008). Rice uptake and recovery of nitrogen with different methods of applying 15N-labeled chicken manure and ammonium sulfate. Plant Production Sci. 11 (3), 271–277. doi: 10.1626/pps.11.271

[B52] Londo J. P. Chiang Y.-C. Hung K.-H. Chiang T.-Y. Schaal B. A. (2006). Phylogeography of Asian wild rice, Oryza rufipogon, reveals multiple independent domestications of cultivated rice, Oryza sativa. Proc. Natl. Acad. Sci. United States America 103 (25), 9578–9583. doi: 10.1073/pnas.0603152103 PMC1480449 16766658

[B53] Mahboob W. Yang G. Irfan M. (2023). Crop nitrogen (N) utilization mechanism and strategies to improve N use efficiency. Acta Physiol Plantarum 45 (4), 52. doi: 10.1007/s11738-023-03527-6

[B54] MAPA. (2017). Ministerio de Agricultura, Pesca y Alimentación. Arroz. Available at: https://www.mapa.gob.es/es/agricultura/temas/producciones-agricolas/cultivos-herbaceos/arroz/.

[B55] MAPA. (2022). Ministerio de Agricultura, Pesca y Alimentación. Ganadería. Available at: https://www.mapa.gob.es/es/ganaderia/temas/default.aspx.

[B56] METEOCAT. (2023). Servei Meteorològic de Catalunya. Climatologies comarcals. Available at: https://www.meteo.cat/wpweb/climatologia/el-clima/climatologies-comarcals/.

[B57] Mileva N. Mecklenburg S. Gascon F. (2018). “New tool for spatiotemporal image fusion in remote sensing—A case study approach using Sentinel-2 and Sentinel-3 data,” in Image and Signal Processing for Remote Sensing Xxiv, vol. 10789 . Eds. Bruzzone L. Bovolo F. (USA: SPIE). doi: 10.1117/12.2327091

[B58] Moeller K. Mueller T. (2012). Effects of anaerobic digestion on digestate nutrient availability and crop growth: A review. Eng. Life Sci. 12 (3), 242–257. doi: 10.1002/elsc.201100085

[B59] Moreno-García B. Guillén M. Quílez D. (2017). Response of paddy rice to fertilization with pig slurry in northeast Spain: Strategies to optimise nitrogen use efficiency. Field Crops Res. 208, 44–54. doi: 10.1016/j.fcr.2017.01.023

[B60] Mutters R. G. Greer C. A. Horwath W. R. (2010). Rice Nutrient Management in California (USA: UCANR Publications). Available at: https://agronomy-rice.ucdavis.edu/ucanr-publications.

[B61] Mzid N. Cantore V. De Mastro G. Albrizio R. Sellami M. H. Todorovic M. (2020). The application of ground-based and satellite remote sensing for estimation of bio-physiological parameters of wheat grown under different water regimes. Water 12 (8), 2095. doi: 10.3390/w12082095. Article 8.

[B62] Nishikawa T. Li K. Inamura T. (2014). Nitrogen uptake by the rice plant and changes in the soil chemical properties in the paddy rice field during yearly application of anaerobically-digested manure for seven years. Plant Production Sci. 17 (3), 237–244. doi: 10.1626/pps.17.237

[B63] Olsen S. R. (1954). Estimation of available phosphorus in soils by extraction with sodium bicarbonate (Issue 939) (USA: US Department of Agriculture). Available at: https://anlab.ucdavis.edu/analysis/Soils/340.

[B64] Padhan B. K. Sathee L. Kumar S. Chinnusamy V. Kumar A. (2023). Variation in nitrogen partitioning and reproductive stage nitrogen remobilization determines nitrogen grain production efficiency (NUEg) in diverse rice genotypes under varying nitrogen supply. Front. Plant Sci. 14. doi: 10.3389/fpls.2023.1093581 PMC10020356 36938028

[B65] Pan G. Zhou P. Li Z. Smith P. Li L. Qiu D.. (2009). Combined inorganic/organic fertilization enhances N efficiency and increases rice productivity through organic carbon accumulation in a rice paddy from the Tai Lake region, China. Agric. Ecosyst. Environ. 131 (3–4), 274–280. doi: 10.1016/j.agee.2009.01.020

[B66] Peng S. Buresh R. J. Huang J. Yang J. Zou Y. Zhong X.. (2006). Strategies for overcoming low agronomic nitrogen use efficiency in irrigated rice systems in China. Field Crops Res. 96 (1), 37–47. doi: 10.1016/j.fcr.2005.05.004

[B67] Pettorelli N. Ryan S. Mueller T. Bunnefeld N. Jędrzejewska B. Lima M. (2013). The Normalized Difference Vegetation Index (NDVI): unforeseen successes in animal ecology. Climate Research 46, 15–27.

[B68] Phongchanmixay S. Bounyavong B. Khanthavong P. Khanthavong T. Ikeura H. Matsumoto N.. (2019). Rice plant growth and nutrient leaching under different patterns of split chemical fertilization on sandy soil using a pot. Paddy Water Environ. 17 (2), 91–99. doi: 10.1007/s10333-019-00701-w

[B69] Pinto R. S. Lopes M. S. Collins N. C. Reynolds M. P. (2016). Modelling and genetic dissection of staygreen under heat stress. TAG. Theoretical and Applied Genetics. Theoretische Und Angewandte Genetik 129 (11), 2055–2074. doi: 10.1007/s00122-016-2757-4 27545985 PMC5069319

[B70] Ponnamperuma F. N. (1972). The chemistry of submerged soils. Advances in Agronomy 24, 29–96. doi: 10.1016/S0065-2113(08)60633-1

[B71] Qaswar M. Yiren L. Jing H. Kaillou L. Mudasir M. Zhenzhen L.. (2020). Soil nutrients and heavy metal availability under long-term combined application of swine manure and synthetic fertilizers in acidic paddy soil. J. Soils Sediments 20 (4), 2093–2106. doi: 10.1007/s11368-020-02576-5

[B72] QGIS Development Team. (2022). QGIS geographic information System; v 3.28.2; open source geospatial foundation project.. Available at: https://qgis.org/en/site/. Accessed on March 30th 2023.

[B73] Qiao J. Yang L. Yan T. Xue F. Zhao D. (2013). Rice dry matter and nitrogen accumulation, soil mineral N around root and N leaching, with increasing application rates of fertilizer. Eur. J. Agron. 49, 93–103. doi: 10.1016/j.eja.2013.03.008

[B74] Qiu H. Yang S. Jiang Z. Xu Y. Jiao X. (2022). Effect of irrigation and fertilizer management on rice yield and nitrogen loss: A meta-analysis. Plants 11 (13), 1690. doi: 10.3390/plants11131690. Article 13. 35807642 PMC9268946

[B75] Rathnayake D. Schmidt H.-P. Leifeld J. Mayer J. Epper C. A. Bucheli T. D.. (2023). Biochar from animal manure: A critical assessment on technical feasibility, economic viability, and ecological impact. Global Change Biol. Bioenergy 15 (9), 1078–1104. doi: 10.1111/gcbb.13082

[B76] Rehman H. Ali I. Ali F. Awan M. I. Wakeel A. Farooq M.. (2023). Nitrogen management strategies to improve crop performance, recovery efficiency and their relationship with physiological indices in dry direct-seeded rice. Int. J. Plant Production 17 (2), 297–308. doi: 10.1007/s42106-023-00239-2

[B77] Rehman T. H. Reis A. F. B. Akbar N. Linquist B. A. (2019). Use of normalized difference vegetation index to assess N status and predict grain yield in rice. Agron. J. 111 (6), 2889–2898. doi: 10.2134/agronj2019.03.0217

[B78] Reimer M. Oelofse M. Müller-Stöver D. Möller K. Bünemann E. K. Bianchi S.. (2023). Sustainable growth of organic farming in the EU requires a rethink of nutrient supply. Nutrient Cycling Agroecosystems. doi: 10.1007/s10705-023-10297-7

[B79] Rouse J. W. Haas R. H. Schell J. A. Deering D. W. (1973). “Monitoring vegetation systems in the great plains with ERTS,” in Third Earth Resources Technology Satellite-1 Symposium-Volume I: Technical Presentations. Eds. Freden S. C. Mercanti E. P. Becker M. A. (Washington, D.C.: NASA), 309–317.

[B80] Salgueiro Romero L. Marcello J. Vilaplana V. (2020). Super-resolution of sentinel-2 imagery using generative adversarial networks. Remote Sens. 12 (15), 2424. doi: 10.3390/rs12152424

[B81] San Bautista A. Fita D. Franch B. Castineira-Ibanez S. Arizo P. Sanchez-Torres M. J.. (2022). Crop monitoring strategy based on remote sensing data (Sentinel-2 and planet), study case in a rice field after applying glycinebetaine. Agronomy-Basel 12 (3), 708. doi: 10.3390/agronomy12030708

[B82] SAS Institute Inc (2020–2021a). Discovering JMP® 16 (NC: SAS Institute Inc: Cary).

[B83] Schmidt F. Knoblauch R. (2020). Extended use of poultry manure as a nutrient source for flood-irrigated rice crop. Pesquisa Agropecuária Bras. 55, e00708. doi: 10.1590/s1678-3921.pab2020.v55.00708

[B84] Segarra J. Buchaillot M. L. Araus J. L. Kefauver S. C. (2020). Remote sensing for precision agriculture: sentinel-2 improved features and applications. Agronomy 10 (5), 641. doi: 10.3390/agronomy10050641. Article 5.

[B85] Singh B.-. (2018). Are nitrogen fertilizers deleterious to soil health? Agronomy 8 (4), 48. doi: 10.3390/agronomy8040048. Article 4.

[B86] Somaweera K.A. T.N. Suriyagoda L. D. B. Sirisena D. N. De Costa W. (2016). Accumulation and partitioning of biomass, nitrogen, phosphorus and potassium among different tissues during the life cycle of rice grown under different water management regimes. Plant Soil 401 (1–2), 169–183. doi: 10.1007/s11104-015-2541-2

[B87] Su N. Xie G. Mao Z. Li Q. Chang T. Zhang Y.. (2023). The effectiveness of eight-years phosphorus reducing inputs on double cropping paddy: Insights into productivity and soil-plant phosphorus trade-off. Sci. Total Environ. 866, 161429. doi: 10.1016/j.scitotenv.2023.161429 36623670

[B88] Thanh T. N. Sasaki Y. Aizawa M. Kakuda K. Fujii H. (2023). Potassium balance in paddy fields under conventional rice straw recycling versus cow dung compost application in mixed crop-livestock systems in Japan. Soil Sci. Plant Nutr. 69 (1), 36–44. doi: 10.1080/00380768.2022.2141052

[B89] Tittebrand A. Spank U. Bernhofer C. H. (2009). Comparison of satellite- and ground-based NDVI above different land-use types. Theor. Appl. Climatol 98 (1), 171–186. doi: 10.1007/s00704-009-0103-3

[B90] Tu C. Li T. Liu X. (2019). “Genetic and epigenetic regulatory mechanism of rice panicle development,” in 2018 International Conference On Biotechnology And Bioengineering (8TH ICBB), vol. 2079 . Eds. Trincone A. Gong M. Hungary: Asia Pacific Assoc Sci Engn & Technol; Chinese Journal Biologicals; Szent Istvan Univ; Hungarian Acad Sci, Ctr Agr Res; Polish Acad Sci, Inst Bioorgan Chem.

[B91] Uga Y. Ebana K. Abe J. Morita S. Okuno K. Yano M. (2009). Variation in root morphology and anatomy among accessions of cultivated rice (Oryza sativa L.) with different genetic backgrounds. Breed. Sci. 59 (1), 87–93. doi: 10.1270/jsbbs.59.87

[B92] Uga Y. Sugimoto K. Ogawa S. Rane J. Ishitani M. Hara N.. (2013). Control of root system architecture by DEEPER ROOTING 1 increases rice yield under drought conditions. Nat. Genet. 45 (9), 1097–1102. doi: 10.1038/ng.2725. Article 9. 23913002

[B93] UNSD. (2021). “United nations statistics division,” in Demographic and Social Statistics (USA). Available at: https://unstats.un.org/unsd/demographic-social/products/dyb/dyb_2021/.

[B94] Verma P. K. Verma S. Pandey N. (2022). Root system architecture in rice: Impacts of genes, phytohormones and root microbiota. Biotech. 12 (9), 239. doi: 10.1007/s13205-022-03299-9 PMC9395555 36016841

[B95] Vermote E. Justice C. Claverie M. Franch B. (2016). Preliminary analysis of the performance of the Landsat 8/OLI land surface reflectance product. Remote Sens. Environ. 185 (2), 46–56. doi: 10.1016/j.rse.2016.04.008 PMC6999666 32020955

[B96] Walkley A. Black I. A. (1934). An examination of the Degtjareff method for determining soil organic matter, and a proposed modification of the chromic acid titration method. Soil Sci. 37 (1), 29–38. doi: 10.1097/00010694-193401000-00003

[B97] Wan Y. Huang Q. Wang Q. Yu Y. Su D. Qiao Y.. (2020). Accumulation and bioavailability of heavy metals in an acid soil and their uptake by paddy rice under continuous application of chicken and swine manure. J. Hazardous Mater 384, 121293. doi: 10.1016/j.jhazmat.2019.121293 31606704

[B98] Wang Y. Lu J. Ren T. Hussain S. Guo C. Wang S.. (2017). Effects of nitrogen and tiller type on grain yield and physiological responses in rice. AoB Plants 9 (2), plx012. doi: 10.1093/aobpla/plx012 28533895 PMC5420812

[B99] Wang S. Wang F. Gao S. Wang X. (2016). Heavy metal accumulation in different rice cultivars as influenced by foliar application of nano-silicon. Water Air Soil pollut. 227 (7), 228. doi: 10.1007/s11270-016-2928-6

[B100] Wei B. Yu J. Cao Z. Meng M. Yang L. Chen Q. (2020). The availability and accumulation of heavy metals in greenhouse soils associated with intensive fertilizer application. Int J Environ Res Public Health 2020 17, 5359. doi: 10.3390/ijerph17155359 PMC7432447 32722363

[B101] Xiao H. -Y. Jiang S. -Y. Wu D. -S. Zhou W. B (2011). Risk element (As, Cd, Cu, Pb, and Zn) contamination of soils and edible vegetables in the vicinity of Guixi smelter, South China. South China. Soil Sediment Contam. 20, 592–604. doi: 10.1080/15320383.2011.587047

[B102] Xie W. Wang G. Zhang Q. Guo H. (2007). Effects of nitrogen fertilization strategies on nitrogen use efficiency in physiology, recovery, and agronomy and redistribution of dry matter accumulation and nitrogen accumulation in two typical rice cultivars in Zhejiang, China. J. Zhejiang University Science B 8 (3), 208–216. doi: 10.1631/jzus.2007.B0208 17323433 PMC1810387

[B103] Zha M. Zhao Y. Wang Y. Chen B. Tan Z. (2022). Strigolactones and cytokinin interaction in buds in the control of rice tillering. Front. Plant Sci. 13. doi: 10.3389/fpls.2022.837136 PMC9286680 35845690

[B104] Zhang X. Fang Q. Zhang T. Ma W. Velthof G. L. Hou Y.. (2020). Benefits and trade-offs of replacing synthetic fertilizers by animal manures in crop production in China: A meta-analysis. Global Change Biol. 26 (2), 888–900. doi: 10.1111/gcb.14826 31495039

[B105] Zhao X. Cai S. Yang B. Zhao H. Zeng K. Fan P.. (2023). Soil nitrogen dynamics drive regional variation in nitrogen use efficiency in rice: A multi-scale study. Eur. J. Soil Sci. 74 (2), e13352. doi: 10.1111/ejss.13352

[B106] Zhao F.-J. Wang P. (2020). Arsenic and cadmium accumulation in rice and mitigation strategies. Plant Soil 446 (1), 1–21. doi: 10.1007/s11104-019-04374-6

