# Supplementary material for: Agronomic performance and remote sensing assessment of organic and mineral fertilization in rice fields
Source: Front Plant Sci. 2023 Oct 4;14:1230012. doi: 10.3389/fpls.2023.1230012 (PMC10582757; doi:10.3389/fpls.2023.1230012)
Supplement: Supplementary file 1 [file DataSheet_1.docx]

Supplementary Material

**Agronomic performance and remote sensing assessment of organic and mineral fertilisation in rice fields**

Karen Marti-Jerez^1**^, Mar Català-Forner^1**^, Núria Tomàs^1^, Gemma Murillo^2^, Carlos Ortiz^2^, María José Sánchez-Torres^3^, Andrea Vitali^4^, Marta S. Lopes^5*^

*^1^* *Institute of Agrifood Research and Technology, Amposta, Spain*

*^2^ Ministry of Climate Action, Food and Rural Agenda, Lleida, Spain*

*^3^* *Global Change Unit, Image Processing Laboratory, University of Valencia, Paterna (46980) Valencia, Spain*

*^4^ Rice Research Centre, Strada per Ceretto 4, 27030 Castello d’Agogna, (PV), Italy*

*^5^ Institute of Agrifood Research and Technology, Lleida, Spain*

*Corresponding author: Marta S. Lopes (marta.dasilva@irta.cat)

** These authors contributed equally to the manuscript


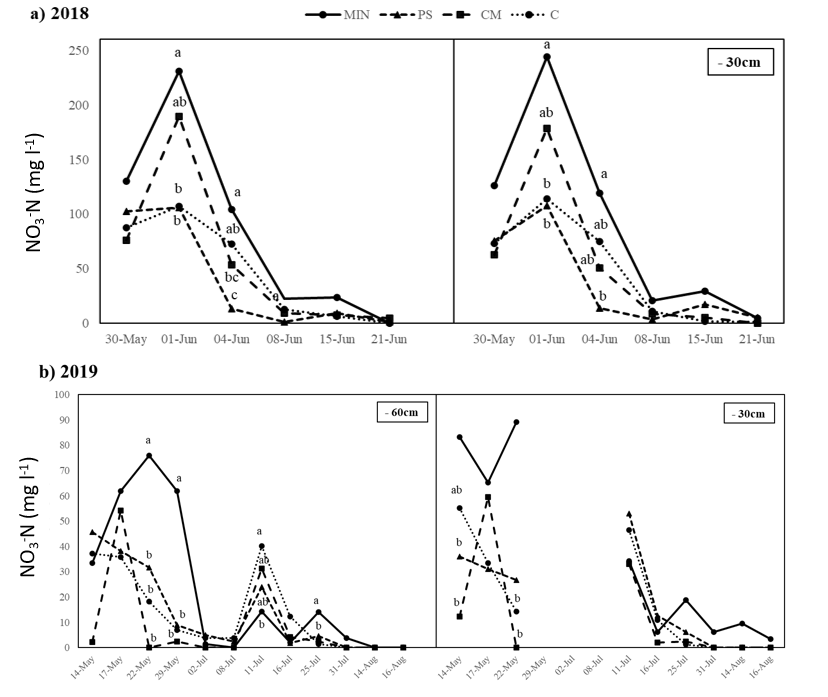


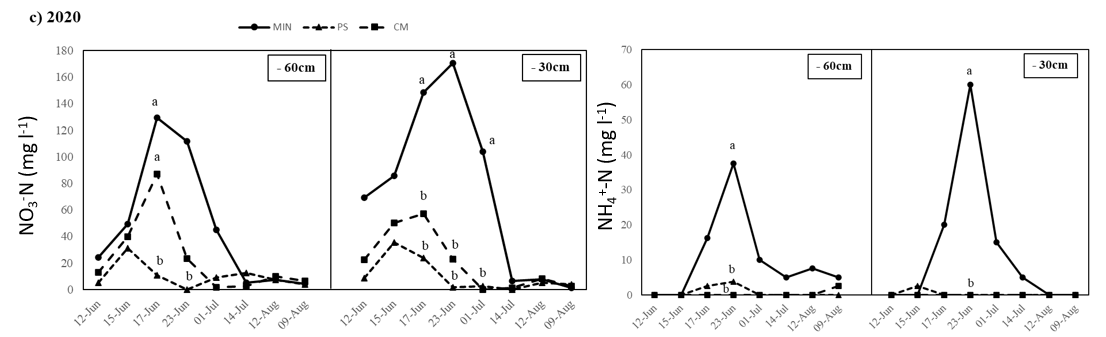


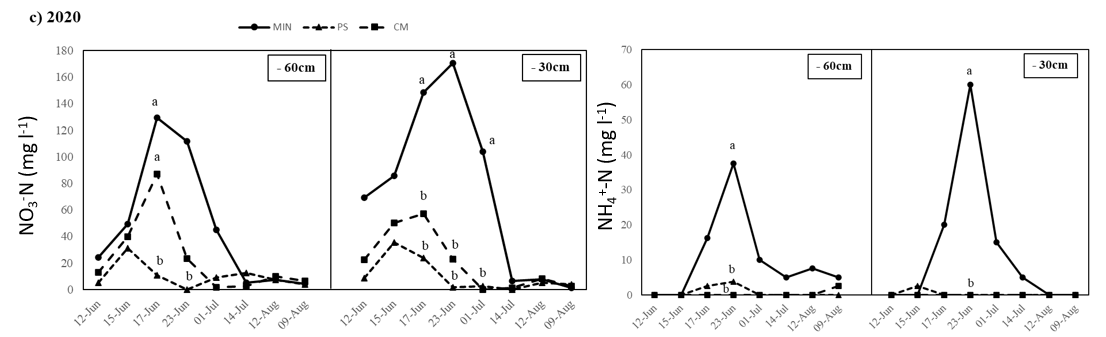


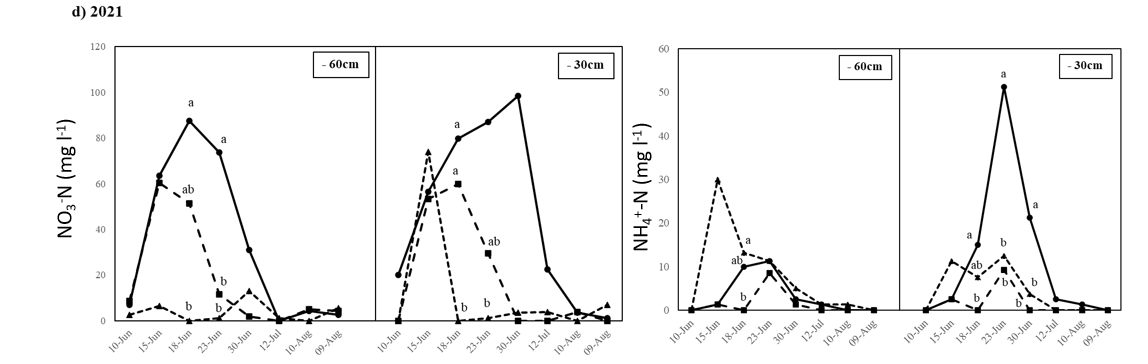


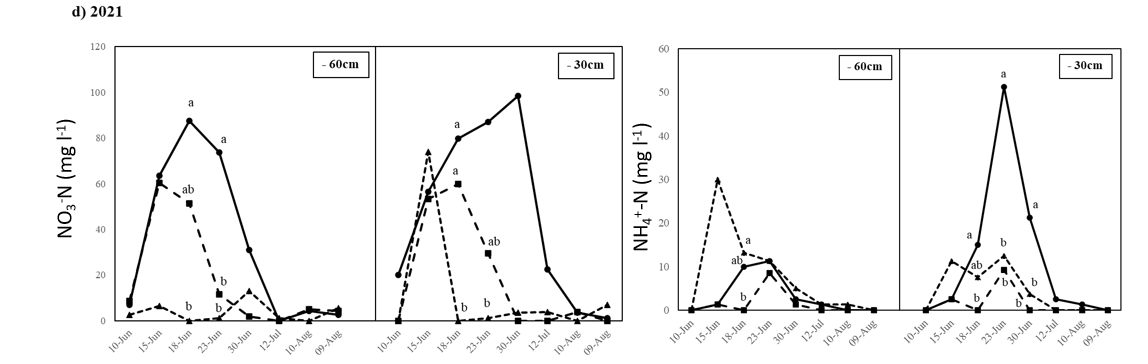


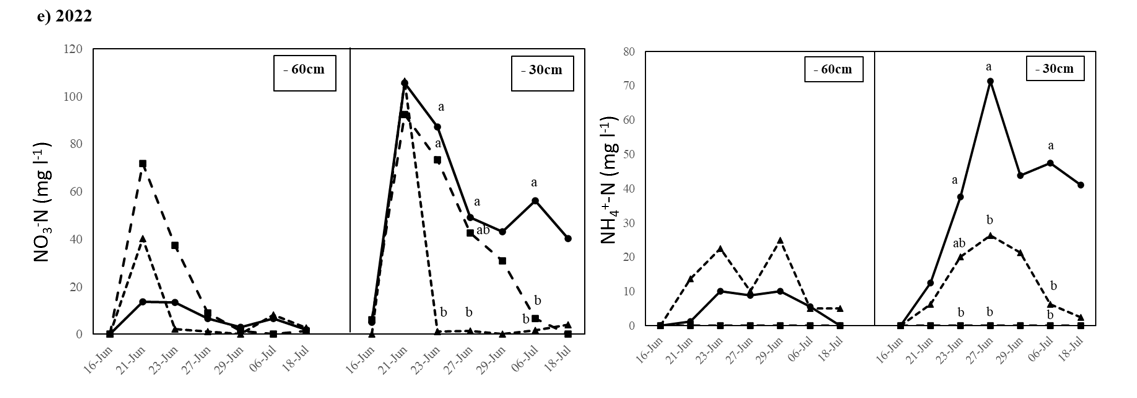


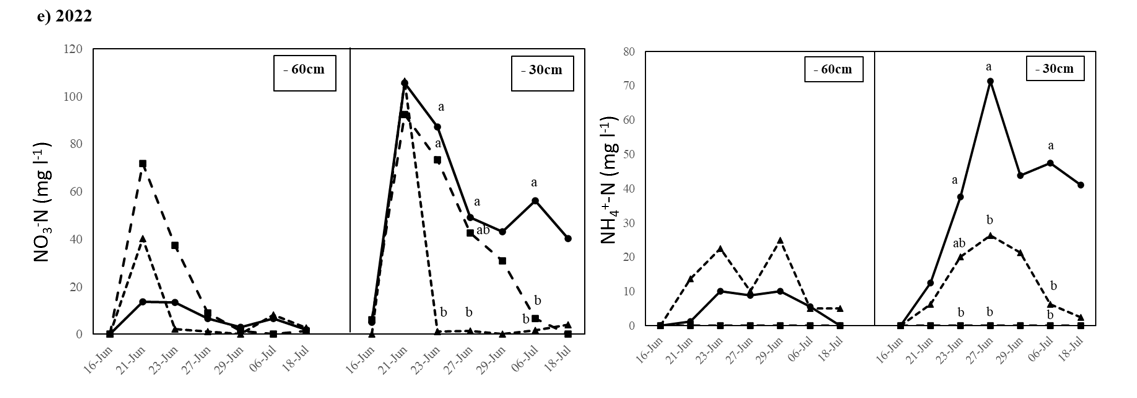


MIN, mineral fertiliser; PS, pig slurry; CM, chicken manure; C, control. Depth: arable land (-30 cm); phreatic water (60 cm). Data were not available for some 2019 dates at -30 cm. The thin lines together with an upper arrow (
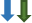
) indicate the flooding + first top-dressing moment (3-4 leaves rice phenological stages) and the second top-dressing moment (panicle initiation rice phenological stages). Different letters indicate significant differences at *p*<0.05, as determined using Tukey’s test (HSD).

***Supplemental Figure 1.*** Water nitrate and ammonium concentration evolution over cultivation time at two different depths (-60 cm and -30 cm). Nitrate data is available from 2018 to 2022. Ammonium data is available from 2020 to 2022.

Supplementary Table 1. Yearly Variations in Six-Year Yield Components, Agronomic Traits, and Disease Scoring for Different Fertilization Strategies

| Year | Fertilisation | | Yield (Mg ha^-1^) | | | Plant density  (plant m^-2^) | | | Panicle density (panicle m^-2^) | | | Tillering capacity (tiller plant^-1^) | | | Panicle efficiency (yield/panicle) | | | Plant height (cm) | | Pyriculariosis IRRI scale (0-9) | | | Helminthosporiosis IRRI scale (0-9) | | |
| --- | --- | --- | --- | --- | --- | --- | --- | --- | --- | --- | --- | --- | --- | --- | --- | --- | --- | --- | --- | --- | --- | --- | --- | --- | --- |
| 2017 | | MIN | 6.79 | ± 0.42 | a | 44 | ± 3.8 | a | 247 | ± 9.3 | a | | 5.8 | a | 27.7 | a | 68.9 | | ± 0.5 | a | 3.5 | a | | 1.0 | a |
|  | | PS | 5.75 | ± 0.35 | a | 48 | ± 2.9 | a | 228 | ± 18.0 | a | | 4.8 | a | 25.4 | a | 66.6 | | ± 1.3 | a | 4.0 | a | | 1.0 | a |
|  | | CM | 5.86 | ± 0.14 | a | 46 | ± 6.9 | a | 217 | ± 11.1 | a | | 5.0 | a | 27.1 | a | 61.4 | | ± 0.5 | b | 3.0 | a | | 1.0 | a |
|  | | C | 2.38 | ± 0.17 | b | 50 | ± 2.7 | a | 130 | ± 4.8 | b | | 2.6 | b | 18.3 | b | 54.8 | | ± 0.8 | c | 3.0 | a | | 1.0 | a |
|  | |  |  |  |  |  |  |  |  |  |  | |  |  |  |  |  | |  |  |  |  | |  |  |
| 2018 | | MIN | 8.84 | ± 0.28 | a | 98 | ± 8.1 | a | 252 | ± 9.0 | a | | 2.6 | a | 35.2 | ab | 74.7 | | ± 1.0 | a | 4.5 | a | | 2.0 | b |
|  | | PS | 8.00 | ± 0.65 | a | 87 | ± 10.0 | ab | 196 | ± 10.4 | b | | 2.3 | a | 40.7 | a | 66.6 | | ± 0.82 | bc | 1.5 | b | | 3.0 | ab |
|  | | CM | 8.73 | ± 0.26 | a | 87 | ± 5.1 | ab | 221 | ± 3.7 | ab | | 2.6 | a | 39.5 | a | 69.6 | | ± 1.2 | b | 3.0 | ab | | 5.0 | a |
|  | | C | 4.42 | ± 0.34 | b | 63 | ± 2.9 | b | 146 | ± 5.0 | c | | 2.3 | a | 30.3 | b | 63.3 | | ± 1.6 | c | 2.5 | ab | | 3.5 | ab |
|  | |  |  |  |  |  |  |  |  |  |  | |  |  |  |  |  | |  |  |  |  | |  |  |
| 2019 | | MIN | 6.98 | ± 0.53 | a | 272 | ± 19.1 | ab | 282 | ± 12.6 | a | | 1.1 | a | 24.8 | bc | 68.0 | | ± 0.6 | a | 6.5 | a | | 5.0 | b |
|  | | PS | 6.78 | ± 0.07 | a | 233 | ± 22.4 | b | 212 | ± 4.5 | b | | 0.9 | a | 32.0 | a | 63.5 | | ± 0.5 | ab | 5.0 | ab | | 5.0 | b |
|  | | CM | 5.65 | ± 0.34 | a | 312 | ± 17.2 | a | 192 | ± 5.8 | bc | | 0.6 | b | 29.5 | ab | 59.8 | | ± 1.4 | bc | 4.0 | bc | | 6.0 | ab |
|  | | C | 3.72 | ± 0.10 | b | 254 | ± 12.4 | b | 164 | ± 5.7 | c | | 0.6 | b | 22.6 | c | 57.4 | | ± 1.2 | c | 3.0 | c | | 7.0 | a |
|  | |  |  |  |  |  |  |  |  |  |  | |  |  |  |  |  | |  |  |  |  | |  |  |
| 2020 | | MIN | 7.50 | ± 0.23 | a | 134 | ± 3.9 | a | 225 | ± 32.3 | a | | 1.7 | a | 36.4 | a | 69.7 | | ± 3.6 | a | 3.0 | a | | 3.0 | a |
|  | | PS | 6.14 | ± 0.52 | ab | 116 | ± 6.5 | a | 221 | ± 27.5 | a | | 1.9 | a | 29.6 | a | 70.5 | | ± 2.7 | a | 3.5 | a | | 3.0 | a |
|  | | CM | 5.70 | ± 0.53 | b | 116 | ± 14.4 | a | 195 | ± 26.5 | a | | 1.7 | a | 30.7 | a | 69.5 | | ± 1.5 | a | 3.5 | a | | 3.0 | a |
|  | | C | 2.84 | ± 0.22 | c | 102 | ± 12.1 | a | 211 | ± 25.6 | a | | 2.2 | a | 14.4 | b | 68.9 | | ± 1.8 | a | 3.5 | a | | 3.0 | a |
|  | |  |  |  |  |  |  |  |  |  |  | |  |  |  |  |  | |  |  |  |  | |  |  |
| 2021 | | MIN | 8.22 | ± 0.28 | a | 271 | ± 16.3 | a | 313 | ± 5.8 | a | | 1.2 | a | 26.3 | ab | 79.3 | | ± 1.6 | a | 1.0 | a | | 1.0 | b |
|  | | PS | 6.28 | ± 0.06 | b | 258 | ± 7.2 | a | 233 | ± 13.7 | b | | 0.9 | b | 27.3 | ab | 74.4 | | ± 0.33 | b | 1.0 | a | | 1.0 | b |
|  | | CM | 6.28 | ± 0.15 | b | 253 | ± 17.3 | a | 228 | ± 3.5 | b | | 0.9 | b | 27.6 | a | 74.1 | | ± 0.5 | b | 1.5 | a | | 1.0 | b |
|  | | C | 3.64 | ± 0.61 | c | 243 | ± 7.7 | a | 174 | ± 9.4 | c | | 0.7 | b | 20.8 | b | 65.6 | | ± 0.9 | c | 1.0 | a | | 3.0 | a |
|  | |  |  |  |  |  |  |  |  |  |  | |  |  |  |  |  | |  |  |  |  | |  |  |
| 2022 | | MIN | 5.86 | ± 0.39 | a | 255 | ± 6.2 | a | 265.5 | ± 3.9 | a | | 1.0 | a | 22.0 | a | 76.9 | | ± 0.6 | a | 3.5 | a | | 3.0 | a |
|  | | PS | 5.46 | ± 0.30 | a | 223 | ± 14.4 | a | 226.1 | ± 10.5 | b | | 1.0 | a | 24.2 | a | 77.1 | | ± 1.6 | a | 3.5 | a | | 3.0 | a |
|  | | CM | 5.51 | ± 0.39 | a | 254 | ± 13.6 | a | 192.3 | ± 2.7 | c | | 0.8 | b | 28.7 | a | 75.5 | | ± 0.9 | a | 4.0 | a | | 3.0 | a |
|  | | C | 1.90 | ± 0.25 | b | 231 | ± 14.3 | a | 136.8 | ± 3.5 | d | | 0.6 | b | 13.9 | b | 66.0 | | ± 1.3 | b | 3.5 | a | | 3.0 | a |
|  | |  |  |  |  |  |  |  |  |  |  | |  |  |  |  |  | |  |  |  |  | |  |  |

MIN, mineral fertiliser; PS, pig slurry; CM, chicken manure; C, control. Measurements are shown as mean ± Standard Error (SE). Results of two-way (top of table, year and fertilisation) and one-way (fertilisation) ANOVA analysis. Different lowercase letters indicate significant differences at *p<0.05* between treatments (one-way ANOVA), as determined using Tukey’s test (HSD).
